# Self-inhibited State of Venezuelan Equine Encephalitis Virus (VEEV) nsP2 Cysteine Protease: A Crystallographic and Molecular Dynamics Analysis

**DOI:** 10.1016/j.jmb.2023.168012

**Published:** 2023-02-13

**Authors:** Gyula Hoffka, George T. Lountos, Danielle Needle, Alexander Wlodawer, David S. Waugh, József Tőzsér, János András Mótyán

**Affiliations:** 1 Department of Biochemistry and Molecular Biology, Faculty of Medicine, University of Debrecen, Hungary; 2 Doctoral School of Molecular Cell and Immune Biology, University of Debrecen, Debrecen, Hungary; 3 Basic Science Program, Frederick National Laboratory for Cancer Research, Frederick, MD 21702, USA; 4 Center for Structural Biology, National Cancer Institute, Frederick, MD 21702, USA

**Keywords:** Venezuelan equine encephalitis virus, protease, alphavirus, crystallography, molecular dynamics

## Abstract

The Venezuelan equine encephalitis virus (VEEV) belongs to the *Togaviridae* family and is pathogenic to both humans and equines. The VEEV non-structural protein 2 (nsP2) is a cysteine protease (nsP2pro) that processes the polyprotein and thus it is a drug target for inhibitor discovery. The atomic structure of the VEEV nsP2 catalytic domain was previously characterized by both X-ray crystallography and computational studies. A modified nsP2pro harboring a N475A mutation in the N terminus was observed to exhibit an unexpected conformation: the N-terminal residues bind to the active site, mimicking binding of a substrate. The large conformational change of the N terminus was assumed to be induced by the N475A mutation, as N475 has an important role in stabilization of the N terminus and the active site. This conformation was first observed in the N475A mutant, but we also found it while determining a crystal structure of the catalytically active nsP2pro containing the wild-type N475 active site residue and K741A/K767A surface entropy reduction mutations. This suggests that the N475A mutation is not a pre-requisite for self-inhibition. Here, we describe a high resolution (1.46 Å) crystal structure of a truncated nsP2pro (residues 463–785, K741A/K767A) and analyze the structure further by molecular dynamics to study the active and self-inhibited conformations of nsP2pro and its N475A mutant. A comparison of the different conformations of the N-terminal residues sheds a light on the interactions that play an important role in the stabilization of the enzyme.

## Introduction

The Venezuelan equine encephalitis virus (VEEV) belongs to the Group IV (+) viruses and is classified under the Alphavirus genus of the *Togaviridae* family, based on the classification system of the International Virus Taxonomy Committee. The alphaviruses can be further subdivided into the New and Old World alphaviruses. Eastern equine encephalitis virus (EEEV), Venezuelan equine encephalitis virus (VEEV), and Western equine encephalitis virus (WEEV) belong to the New World viruses, whereas Sindbis virus (SINV), Semliki forest virus (SFV), and the Chikungunya virus (CHIKV) belong to the Old World virus group.^1–2^

Infection by the encephalitic alphaviruses such as VEEV can cause mild to severe symptoms in both humans and equines. VEEV infection usually manifests itself as mild, flu-like symptoms, but neurological symptoms also appear in approximately 14% of infected individuals. The case fatality rate is low (~1%).^3^ Multiple viruses, such as VEEV and EEEV, have been classified by the Centers for Disease Control and Prevention (CDC) as potential agents for use in biological weapons due to their high infectivity *via* the aerosol route in both humans and livestock.^4^ Currently, there is a lack of FDA-approved antiviral drugs to treat New World alphavirus infections.^5^ With the continued threat of the emergence of alphaviruses, there is a need for further efforts to develop antiviral agents against VEEV and other alphaviruses.^6–11^

**Figure 1.**
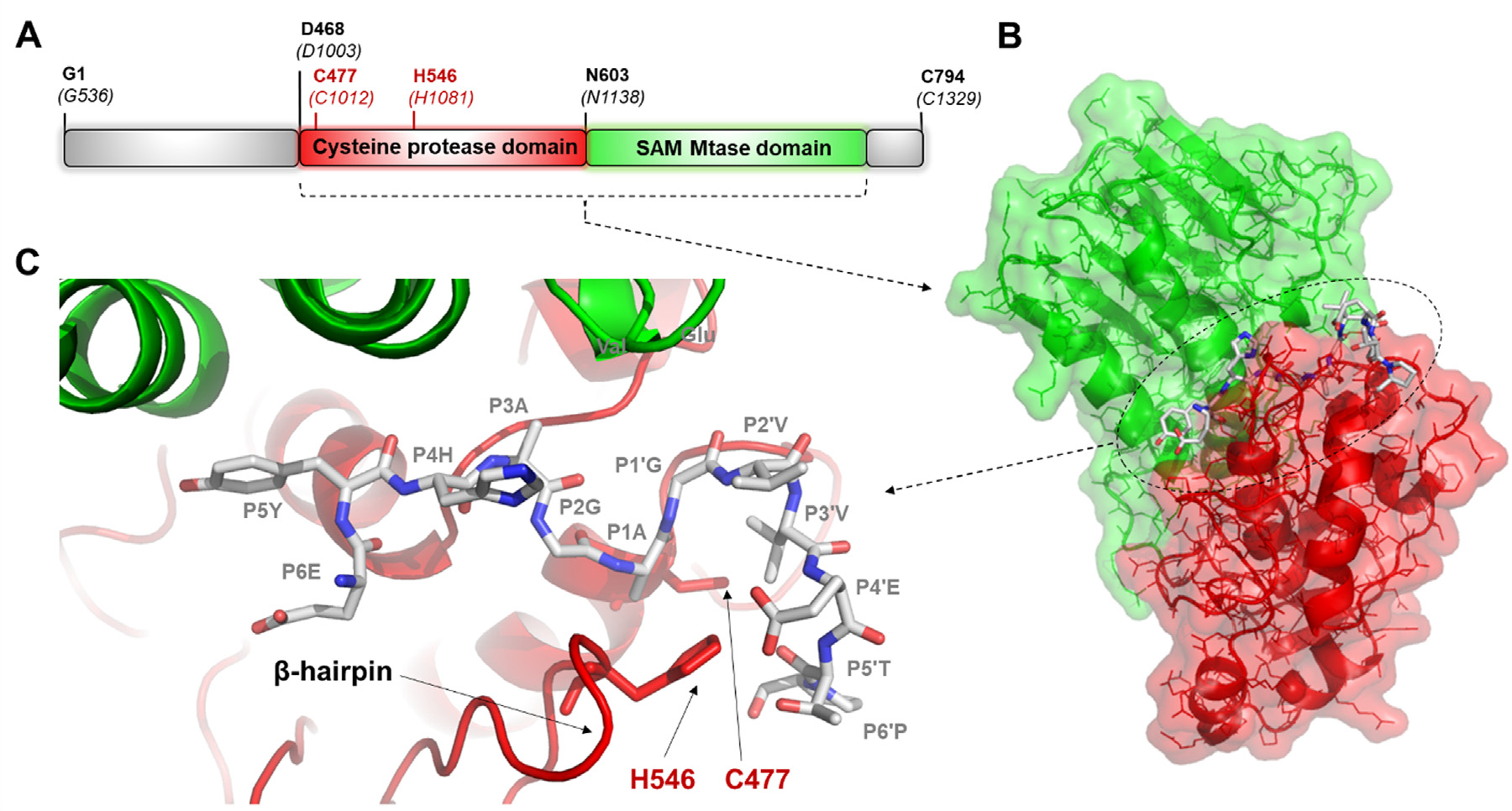
Complex of VEEV nsP2pro and EYHAGAGVVETP oligopeptide substrate. **(a)** The domain organization of the VEEV nsP2pro. Residues are labeled according to the numbering of nsP2 and that of the nsP1234 polyprotein (in parentheses) (UniProt ID: P27282). Aligned sequences of VEEV, SINV and CHIKV polyproteins are shown in Figure S1. (**b**) The overall structure of the protease bound to a peptide substrate is shown based on the enzyme-substrate complex that was modeled previously.^15^ (**c**) Enlarged view of the active site with the modeled position of the EYHAGAGVVETP oligopeptide substrate (P6-P6′ residues).

Alphaviruses have a ss(+) RNA genome that contains two open reading frames (ORFs). The first and second ORFs code for the nonstructural proteins (nsPs) and for the structural proteins, respectively.^12^ The polyprotein that is translated from the first ORF (nsP123 or nsP1234) is proteolytically processed to release the nsP1, nsP2, nsP3, and nsP4 proteins. The VEEV nsP2 contains an N-terminal region of unknown function, a helicase domain, and a cysteine protease domain that is linked to an S-adenosyl-L-methionine-dependent RNA methyltransferease domain (SAM MTase) in its C terminus (Figure 1). The protease (nsP2pro) is responsible for the processing of the precursor via cleavage between the nsPs.

The VEEV nsP2pro is a papain-like cysteine protease. The active enzyme consists of two subdomains; the N-terminal subdomain (which contains the active site residues C477 and H546) and the C-terminal SAM MTase subdomain that lacks enzymatic activity. Both subdomains are involved in the formation of the substrate binding cleft and in substrate recognition. The substrate binds in a cleft between the protease and the SAM MTase subdomains.^13^ Only a few structural coordinates are available currently in the Protein Data Bank (PDB) for alphavirus proteases (Table 1) and none contain a bound substrate. Therefore, enzyme-substrate interactions of VEEV nsP2 have been studied mostly by molecular modeling.^14–15^

Almost all of the available structures represent the active conformation of the enzyme (Table 1), although alternative conformations that were considered to contribute to enzyme inactivation are also found in the PDB for both VEEV and CHIKV. Previously, it has been shown that the N terminus of the VEEV nsP2, which is exposed on the surface in the active (open) conformation, may have an alternative (closed) conformation, in which the N-terminal loop is flipped into the active site, thereby mimicking the binding of a substrate. This self-inhibited conformation was first described for a VEEV nsP2pro construct harboring the N475A mutation that is located near the N terminus.^16^ In this paper, the active and self-inhibited conformations are referred to as they were previously differentiated (A’ and B’), respectively. In addition, the VEEV nsP2pro residues are numbered according to nsP2 numbering (polyprotein numbering is shown in Figure S1). Structures of the CHIKV nsP2pro demonstrated another possible mechanism for blocking the active site which differs from that observed in the inactivation of VEEV nsP2pro. In the CHIKV nsP2pro, binding of the substrate is regulated by a flexible loop (β-hairpin) encompassing the N547 residue. That residue, preceding the catalytic H548, was found to play an important role in the interdomain interactions and contributed to the regulation of substrate binding (these CHIKV nsP2pro residues correspond to the N545 and H546 residues of VEEV nsP2pro, respectively). Accordingly, the N547A mutation was shown to significantly reduce the catalytic efficiency of CHIKV nsP2pro.^17^

The VEEV nsP2pro N475 residue is located in close proximity to the catalytic Cys477. N475 is known to interact with R662 of the SAM MTase domain. Due to the role of N475 in the coordination of substrate binding and nucleophilic attack that happens at the carbonyl carbon of the scissible bond (at the S1 site), its mutation to Ala was found to alter the steady-state kinetic parameters as compared to the wild-type VEEV nsP2pro. The N475A mutant enzyme was found to exhibit a higher K_M_, but reduced k_cat_ and k_cat_/K_M_ values in phosphate buffer at pH 6.0,^14^ and the differences were even more profound in HEPES buffer at pH 7.0.^16^ The melting temperature (T_M_) of the N475A mutant was slightly higher (+2.1 °C) than that of the wild-type.^16^ The CHIKV N475A mutant also showed reduced k_cat_/K_M_ as compared to the wild-type enzyme and the reduced catalytic efficiency suggests that N475 contributes an important function.^17^ The effects of the N475A mutation were considered to be the consequences of the conformational changes of the N terminus.^16^

To study the effects of the N-terminal extension on the protease activity, we designed two forms of VEEV nsP2pro.^15^ The nsP2pro-1 construct consists of residues M457-A792 and lacks the last two C-terminal residues (793–794). The longer nsP2pro-2 construct consists of residues 436–792 and contains a longer N-terminal extension (residues 436–457) compared to nsP2pro-1. We observed that this extension had no substantial effect on the protease activity *in vitro*. While investigating the specificity of VEEV nsP2pro-2 *in vitro*, a remarkable decrease in enzymatic activity was observed after incubation at 30 °C, despite using optimal conditions for time-course protease assays.^15^ Thermal instability has been previously observed for the Semliki forest virus (SFV) nsP2 protease, which showed a decrease in the enzymatic activity after incubation longer than 10 min at 30 °C.^19^ The N terminus of VEEV nsP2pro, encompassing the active site Cys477, is located in the proximity of the active site. Thus, the conformation of this region may be an important determinant of enzymatic activity. Accordingly, a self-inactivated state of the VEEV nsP2pro N475A mutant has been described and the inactive conformation was found to be formed due to the mutation of the N475A residue which destabilized its N terminus.^16^ A clearer understanding of this structure–function relationship is of interest because of the contributions of VEEV nsP2pro to the viral infection-induced phenotype (via processing of the host proteins).^20^

The SAM MTase domain significantly contributes to the proteolytic activity via formation of the substrate-binding cleft.^13^ Besides being necessary for the formation of a functional protease, the SAM MTase domain of SINV was found to also play a role in viral RNA replication and virus-mediated host modulation. These functions are mediated in part by its positively charged surface-exposed residues, as was proved by determining the effects of the alanine-substitutions on plaque phenotypes, temperature sensitivity, RNA synthesis, and proteolytic activity. Although, many of the studied mutations did not interfere with the proteolytic function of nsP2, virus viability, or plaque phenotype, the mutagenesis study helped better understanding of the diverse functions of the SAM MTase domain, and provided useful information for the design of non-cytopathic viral vectors for gene delivery.^21^ The VEEV nsP2pro studied in this work contained K741A and K767A surface-entropy reduction mutations at its SAM MTase domain. The mutated residues are not involved in substrate binding, unlike K705 and K706 residues of this domain at the S4 site.^14^ The K741 and K767 VEEV nsP2 residues correspond to those of the R755 and R781 of SINV nsP2 SAM MTase domain. The R781A SINV mutation was found previously to cause no change of virus viability or plaque phenotype.^21^

Here we report a high-resolution crystal structure of the VEEV nsP2pro, which contains the wild-type N475 residue at the N terminus and K741A/K767A surface entropy reduction mutations in the SAM Mtase domain distant from the active site. We observe two conformations (active and inactive) of the N-terminal extension. Molecular dynamics (MD) simulations were performed to study the conformational and dynamical characteristics of the active site. The interactions of the active site, either with the N-terminal residues or a substrate, were also compared for the enzymes which contain an asparagine (wild-type) or alanine (mutant) residue in 475th position (according to nsP2pro numbering). The structural observations were also compared to those of CHIKV nsP2pro.

## Results

### Crystal structure of VEEV nsP2pro (R463-T785, K741A/K767A)

We determined a high-resolution crystal structure of the VEEV nsP2pro encompassing residues R463-T785. Surface entropy reduction mutations were introduced at residues K741 and K767 of the SAM MTase domain where the lysine residues were mutated to alanine. The goal of this modification was to obtain crystals that would diffract to high resolution for use in ligand soaking experiments and co-crystallization trials. Mutations were not introduced to the cysteine protease domain. The construct harboring the K741A/K767A mutations yielded crystals that diffracted to 1.46 A resolution. X-ray data collection and refinement statistics are shown in Table 2. The refined structure exhibited a final R_work_ of 0.154 and final R_free_ value of 0.197.

**Figure 2.**
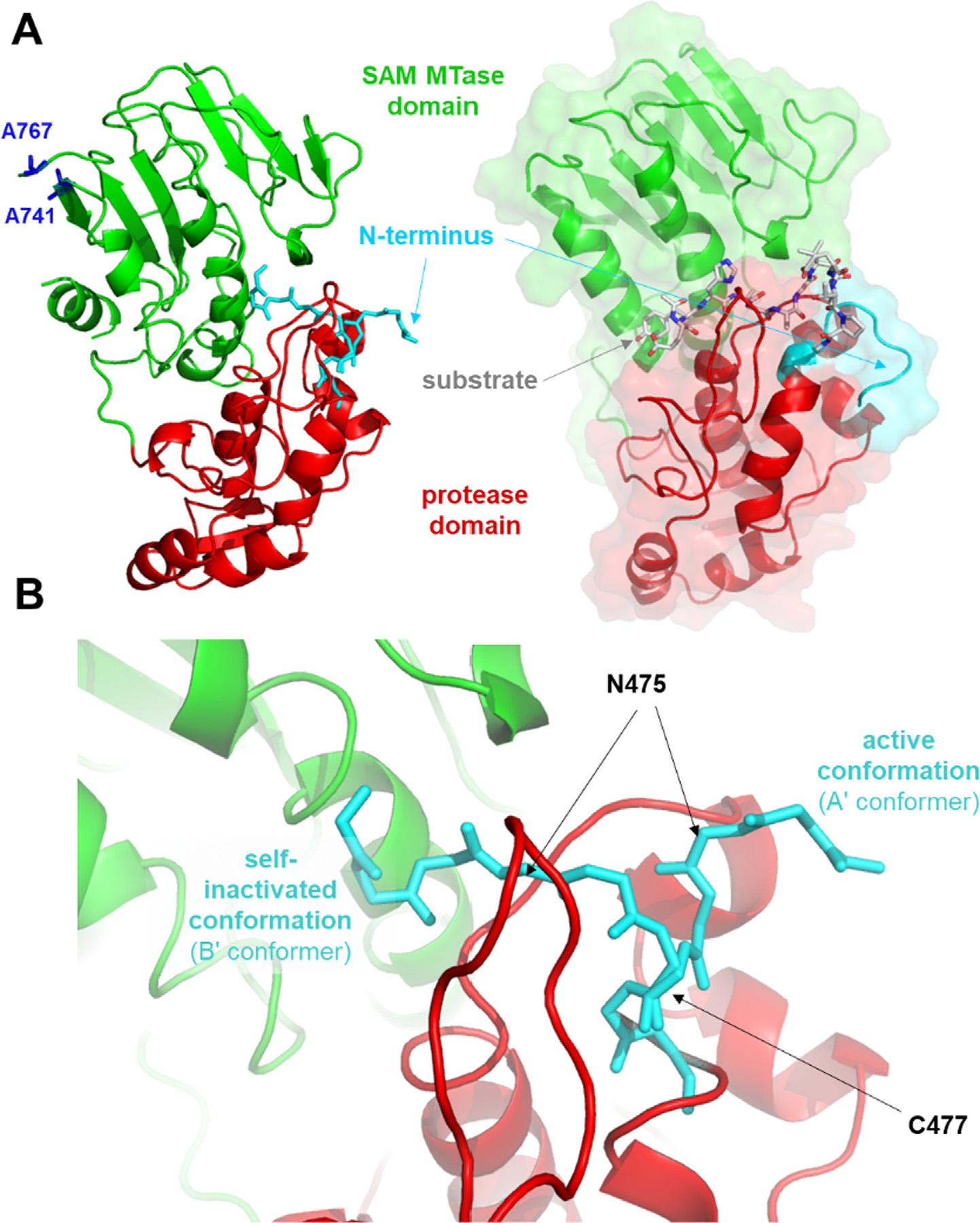
Overall structure and active site of the K741A/K767A mutant VEEV nsP2pro. **(a)** The active and self-inhibited conformations of the K741A/K767A mutant VEEV nsP2pro (PDB code: 8DUF) are shown. For comparison, the modeled complex of the protease and the EYHAGAGVVETP oligopeptide substrate (P6-P6′ residues) is also shown.^15^ (**b**) Enlarged view of the active site showing the different conformational states of the N terminus. The main chain atoms of the ${\;}^{472}{AKANVCW}^{478}$ residues are shown.

Inspection of the electron density maps revealed the presence of two conformations of the N-terminal oligopeptide residues prior to the catalytic C477. Electron density for the N-terminal residues was visible starting with K473 of the A’ (active, ~45% occupancy) conformer and N472 of the B’ (self-inhibited, ~55% occupancy) conformer. Although the side chain is not visible for N472 in the latter conformer, the side chain density is well defined for residues 473–476 (Figure S2). By comparison, the refined occupancies of the N terminus in conformations A’ and B’ were approximately 40 and 60%, respectively, in the case of the N475A mutant VEEV nsP2.^16^ No electron density was visible for residues K769-K775 in the C-terminal region and they were not modeled, but the electron density for the remainder of the C terminus was visible through T785. In the A’ (open) conformation, the N-terminal loop is exposed on the enzyme surface and the active site is accessible to substrate and ligands. In contrast, the B’ (closed) conformation reveals that the N-terminal residues ^472^AKANVC^477^ are flipped into the active site and to the interdomain cleft beneath the β-hairpin, thus acting as a pseudo-substrate (Figure 2). In the B’-conformer, the N-terminal residues occupy the S5-S1′ substrate binding site and the nucleophilic C477 residue in bound to the S1′ site (nomenclature according to Schechter and Berger.^22^

The structure of the N475A nsP2pro showed no self-processing for the B’ conformation.^16^ The B’ conformer of the K741A/K767A mutant nsP2pro construct used in our study was considered to be catalytically incompetent because the density for ^472^AKANVC^477^ is continuous and of high quality (Figure S2).

### Comparison of nsP2pro structures

**Figure 3.**
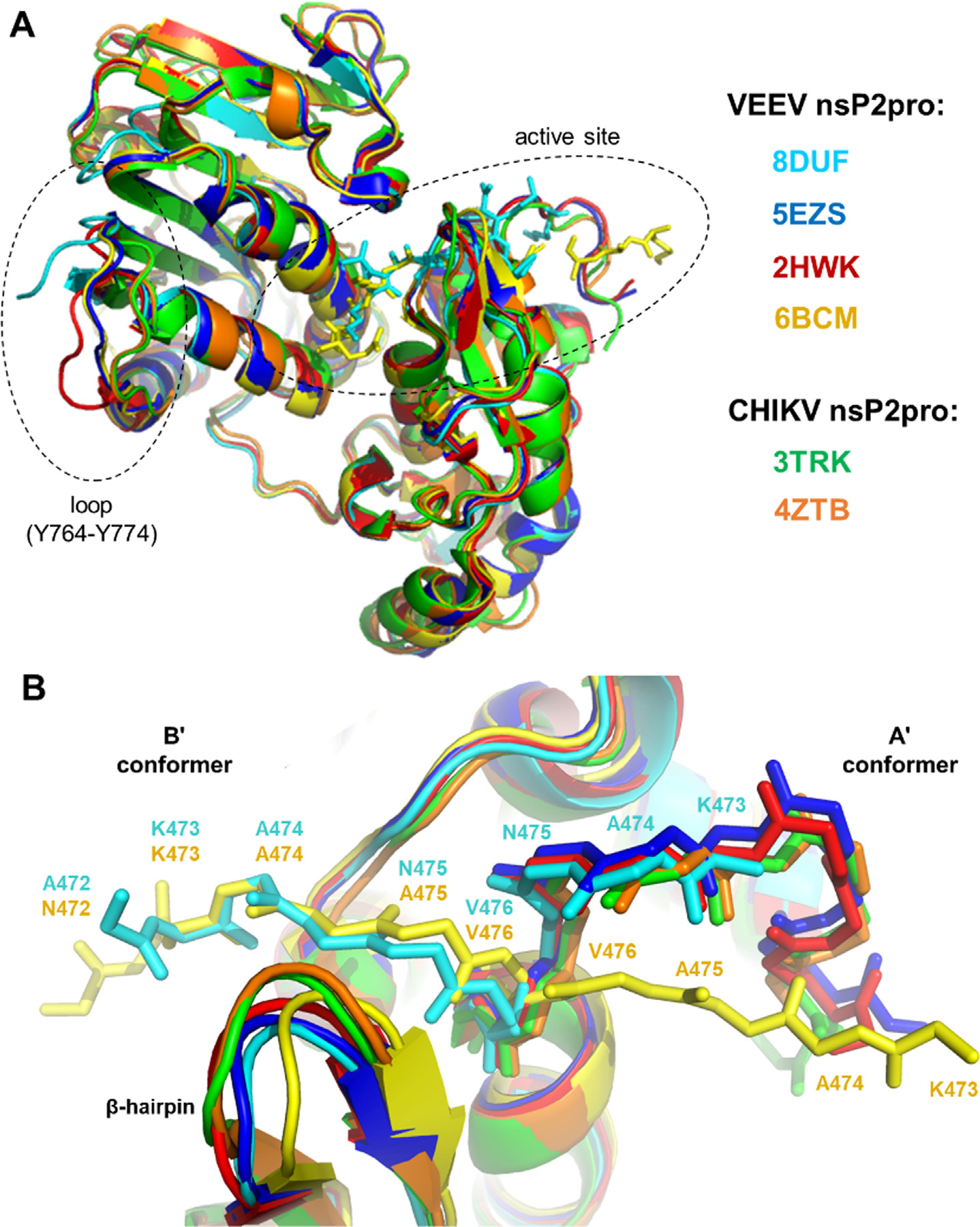
Comparison of nsP2pro structures. **(a)** Alignment of overall nsP2pro structures. The different structures are shown by different colors, the color code is indicated in the figure. The active site and the loop for which the electron density was visible only in part are circled by dashed lines. (**b**) Comparison of N termini in the A’ (active) and B’ (self-inactivated) conformers. The backbone of 472–477 sequence motif is shown by sticks, the residues are labeled.

The overall structures of VEEV and CHIKV nsP2 are highly similar. Most available crystal structures exhibit the A’ conformation of the N-terminal residues that are exposed to the surface, thereby keeping the active site open and accessible for substrate binding (Figure 3(a)). The spatial arrangement of the N terminus is highly similar in the case of the VEEV nsP2 enzymes that have the active conformation (PDB IDs: 2HWK, 5EZS, 5EZQ, 8DUF), whereas the N terminus of the N475A mutant (PDB ID: 6BCM) does not overlay with the previously described wild-type VEEV nsP2 structures.^16^ Here, we found that the conformations at the N termini of the K741A/K767A mutant VEEV nsP2 (PDB ID: 8DUF) and the N475A mutant (PDB ID: 6BCM) are highly similar in the self-inhibited state. The A’ conformation of the N terminus (473–476) of the K741A/K767A mutant VEEV nsP2 (PDB ID: 8DUF) is significantly different from that of the N475A mutant (PDB ID: 6BCM). This comparison indicates that, while the N475A mutation alters the conformation of the N terminus in the A’ conformer, the N-terminal loop can flip into the active site and act as a pseudo-substrate in the enzyme that contains the wild-type N475 residue as well (adopting the B’ conformation).

The SAM MTase domain of the crystallized VEEV nsP2pro (PDB ID: 8DUF) contained K741A and K767A surface entropy reduction mutations. The possible effects of these amino acid substitutions on the loop encompassing Y764-Y774 residues were estimated based on the comparison of crystal structures. Although, the electron density was not visible for K769-K775 residues in the C-terminal region of VEEV nsP2pro, the spatial conformation of the visible Y764-A768 region is also slightly different as compared to other structures lacking these surface mutations (PDB ID: 6BCM, 2HWK and 5EZQ) (Figure 3(a), Figure S3). This difference imply that the Y764-Y774 loop potentially has an increased flexibility upon these simultaneous mutations. Other regions of the nsP2pro’s aligned well and showed no considerable conformational differences, indicating no remarkable distant effects of K741A and K767A mutations on the protease domain.

### β-hairpin and N545 residue

**Figure 4.**
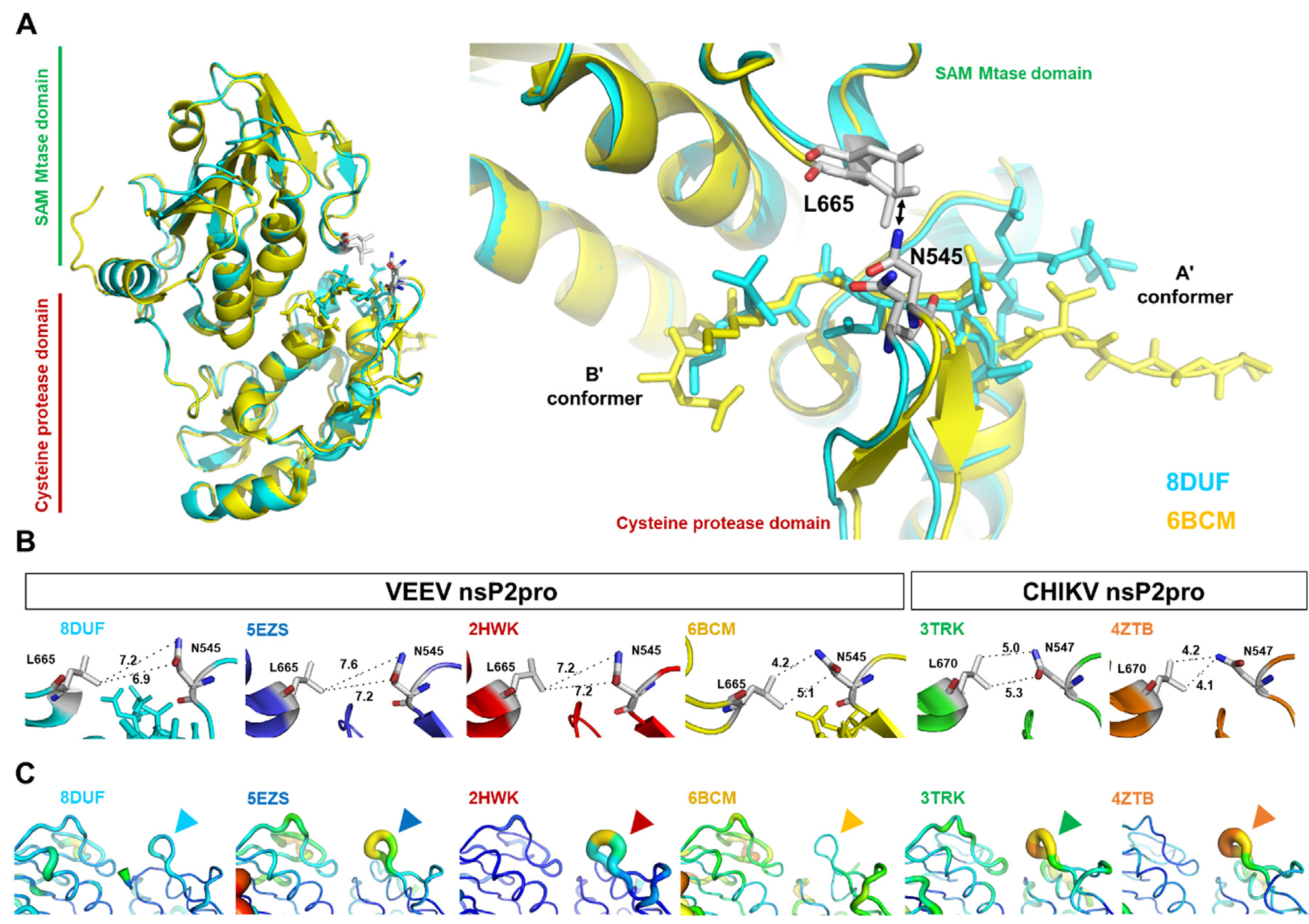
Comparison of subdomain interfaces in nsP2pro crystal structures. **(a)** Overall structure of VEEV nsP2pro representing the A’ and B’ conformers, the L665 and N545 residues belonging to the respective SAM MTase and protease domains are labeled (according to VEEV nsP2pro numbering). The arrow indicates the distances between the residues shown in figure part B. (**b**) The interatomic distances (Å) between Asn (in β-hairpin) and Leu (in loop between β7-strand and α9-helix) residues are shown. (**c**) Comparison of B-factors of subdomain interfaces in nsP2pro structures. Arrowheads show the b-hairpins (encompassing N545 and H546 residues, according to VEEV nsP2pro numbering) in VEEV and CHIKV nsP2pro crystal structures.

A crystal structure of the CHIKV nsP2pro (PDB code: 4ZTB) showed that the β-hairpin, which contains the catalytic H546 residue, can block the active site by restricting substrate access due to the short distance (4.1 Å) between N547 and L670.^17^ This distance between the corresponding residues is highly similar in the other CHIKV nsP2pro structure (5.0 A, PDB ID: 3TRK). Interestingly, an almost identical distance has been observed for a self-inactivated structure of the VEEV nsP2 N475A mutant (4.2 Å, PDB ID: 6BCM), but longer interatomic distances can be seen in the active conformations of the wild-type enzymes (PDB IDs: 5EZQ, 5EZS, and 2HWK) as well (Figure 4(a)).

In the crystal structure described here (PDB ID: 8DUF), the interdomain distance is also relatively longer (6.9 Å) and the loops are predicted not to be close enough to prevent binding of the N terminus to the active site (Figure 4(b)). Based on this observation, we assume that the formation of the self-inactivated state of the VEEV nsP2 is not directly connected to the closure of the loops and the close proximity of the loops does not prevent the flip of the N-terminal residues into the substrate binding cleft. The VEEV N475A mutant may be more prone to closure of the loops, as the structure of the mutant enzyme shows a relatively shorter distance compared to the wild-type (N475-containing) structures (Figure 4(b)).

The atomic displacement parameters (ADPs, also called B-factors) also indicate a relatively higher flexibility of the b-hairpin (encompassing the N545 residue) in other structures (PDB IDs: 2HWK, 5EZS, 4ZTB, and 3TRK). Such elevated ADPs were not observed in the structures where the self-inhibited conformations are also formed (PDB IDs: 8DUF and 6BCM).

**Figure 5.**
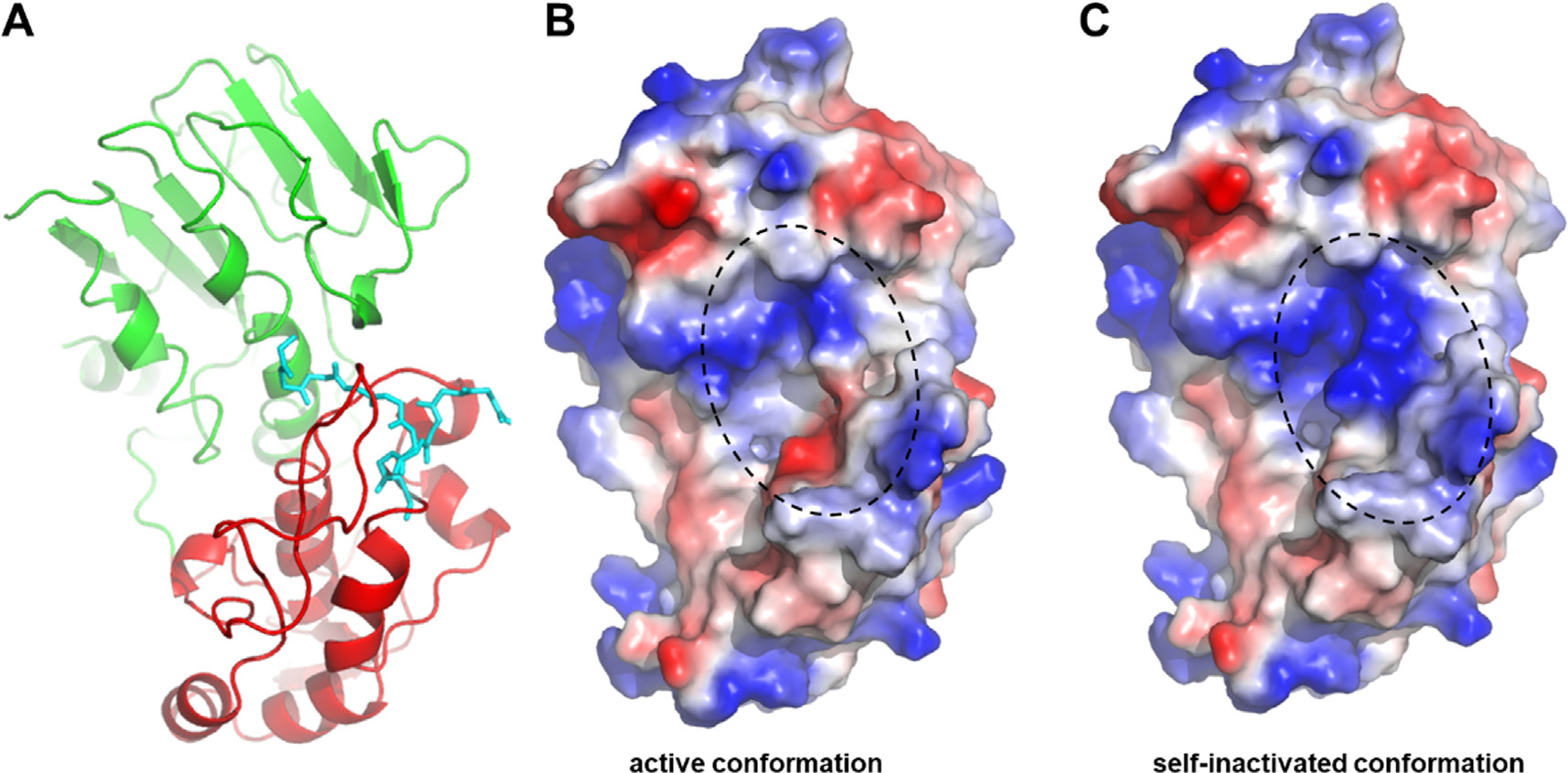
Surface of the K741A/K767A mutant VEEV nsP2pro in the active and self-inactivated state, colored by surface electrostatic potential. The structure of VEEV nsP2pro is shown based on 8DUF.pdb, the structure is shown by ribbon (**a**) and surface representations (**b, c**). The N-terminal residues are shown by cyan sticks, both in the open and closed conformations (**a**). The charge-smoothed surface is shown based on automated qualitative electrostatic representation of PyMOL. Color code of surface electrostatics: blue, basic; red, acidic; and white, neutral. The active site is circled by dashed line (**b, c**).

The distribution of the electrostatic surface potentials was found to be different in the closed and open conformations (Figure 5). In the closed conformation, the size of the area having positive electrostatic potential is larger compared to the open conformation. The relevance of this phenomenon regarding the functional characteristics has not been explored *in vitro* to date, and it remains to be determined whether this region may be more prone to aggregation.

### Molecular dynamics analysis of VEEV nsp2pro

In addition to the determination of the crystal structure, we performed molecular dynamics (MD) computational studies of the available VEEV nsP2pro crystal structures in order to investigate the dynamic properties of the nsP2rpro N terminus and the active site. For MD analysis, we used the coordinate files from the PBD depositions: 8DUF (wild-type residue in the protease domain: N475), 6BCM (N475A mutant), and 2HWK (wild-type). We examined both the active (dyn-act) and the self-inhibited (dyn-inact) forms of 8DUF and 6BCM structures. For comparison, the 2HWK structure (exhibiting only the A’ conformation) was also used.

**Figure 6.**
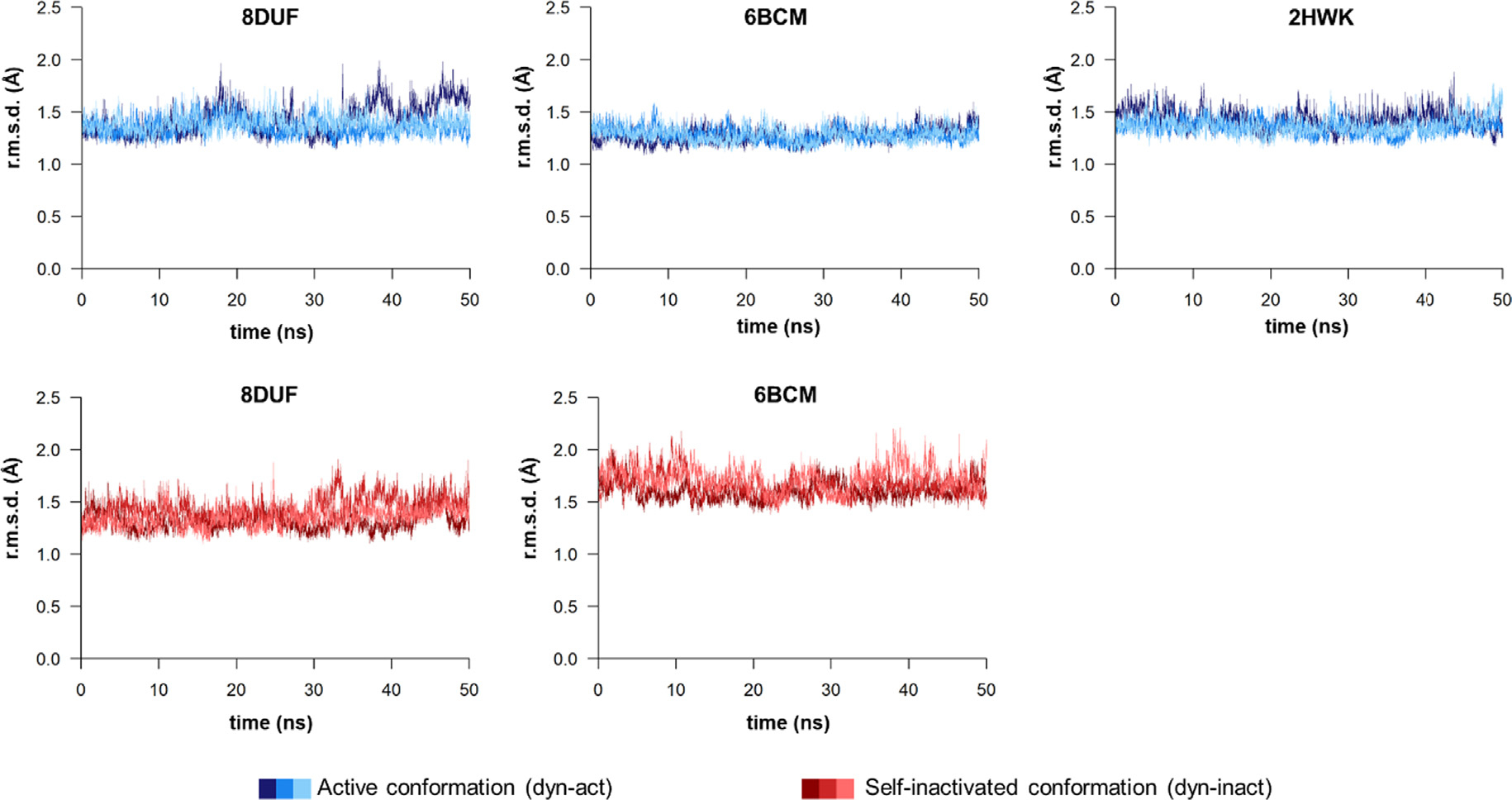
R.m.s.d. values during trajectories. R.m.s.d. values (Å) are shown for the 8DUF, 2HWK and 6BCM. Both main and side chain atoms were included while calculating r.m.s.d. values. Values are colored differentially for the two different enzyme forms (blue and red for dyn-act and dyn-inact, respectively), the different shades represent the results obtained from three individual simulations.

We performed MD simulations to compare the wild-type N475 residue-containing (PDB IDs: 8DUF and 2HWK) and the N475A mutant structures (PDB ID: 6BCM). Three individual simulations were carried out for each structure. The r.m.s.d. values obtained from the simulations showed only moderate overall fluctuations during the trajectories and the three separate individual simulations for each ensemble showed only minimal differences (Figure 6). The r.m.s.d values were comparable in the active and self-inactivated conformations, and were close to 1.5 Å. A slightly higher overall r.m.s.d was observed only for the self-inactivated (B’) conformation of the N475A mutant (6BCM^dyn-inact^) as compared to its active (A’) conformation, indicating an elevated overall flexibility of the structure upon the N475A mutation.

### Interactions of the N terminus

**Figure 7.**
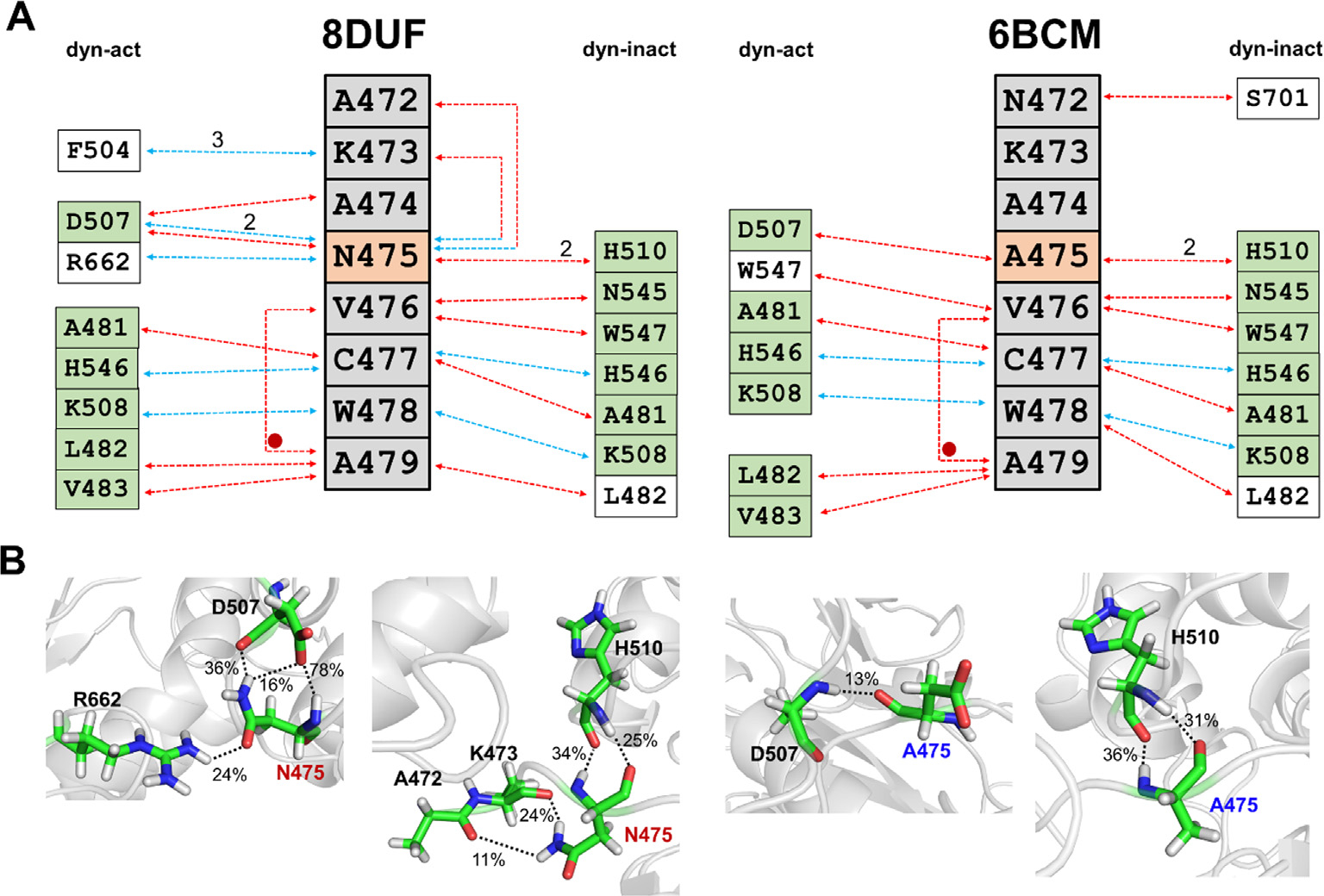
Intramolecular interactions of N-terminal residues. **(a)** Only those H-bond interactions are shown which are present in ≥10% of trajectories. N-terminal residues are shown by grey background, the 475th residue is highlighted by orange. The arrows show interactions between the residues of the active site and the N terminus, in the case of the A’ and B’ conformers. Red and blue arrows indicate backbone- and side chain-mediated H-bonds, respectively to the N-terminal residue. The numbers above the arrows indicate the number of same type of interaction between the residues. The active site residues that form interactions with the same N-terminal residue in A’ (8DUF^dyn-act^ and 6BCM^dyn-act^) and B’ (8DUF^dyn-act^ and 6BCM^dyn-act^) conformers are shown by green background, or are marked with a red circle in the case of residues of the N terminus. (**b**) Representative interactions involving the residue at the 475th position are also shown for each structure. The dashed lines indicate H-bonds, the bonds are labeled by the percentage of the bond being present during the trajectory (if ≥10%).

To investigate the characteristics of the self-inhibited conformation, we mapped the intramolecular interactions of the N terminus (^472^AKANVCWA^479^), both for the A’ and B’ conformers (Figure 7(a)). The contacts of residues 472–477 showed only slight differences, the overall patterns of interactions are similar in the K741A/K767A mutant (PDB ID: 8DUF) and N475A mutant structures (PDB ID: 6BCM) in both conformers. The oppositely charged active site residues (C477 and H546) formed a H-bond interaction in all of the trajectories. Interestingly, in the self-inactivated conformation, both N475 and A475 residue was able to form main chain H-bond interactions with H510, whereas this interaction was not formed in the active conformation. The N475A mutation notably decreased the number of H-bond interactions of this residue, as the apolar side chain is unable form any. The H-bond contacts of the residue 475 are shown in Figure 7 (b).

### β-hairpin and N545 residue

While the β-hairpin showed relatively lower ADPs in both the self-inhibited structures (8DUF and 6BCM) (Figure 4(c)), the trajectories were further analyzed in order to determine the changes of interdomain distances by measuring the distances between the center of mass of N545 and L665 residues, which are located at the subdomain interface (Figure 4).

**Figure 8.**
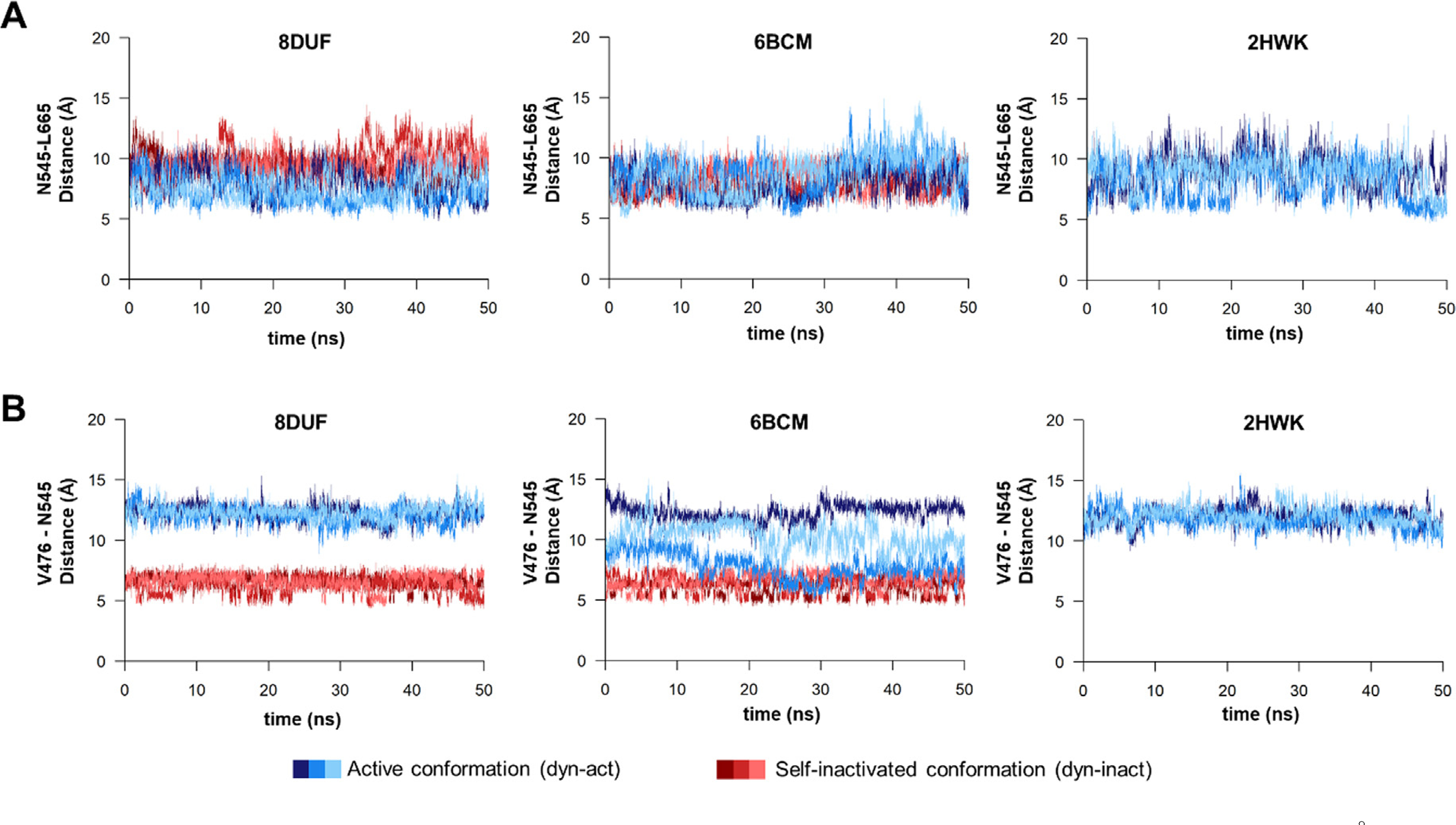
Changes of interdomain distances during trajectories. **(a)** The changes of the distances (Å) between the center of mass of N545 and L665 residues during the trajectories are shown for the 8DUF, 2HWK and 6BCM structures. (**b**) The changes of the distance (A) measured between the center of mass of V476 and N545 residues during the trajectories. Values are colored differentially for the two different enzyme forms (with blue and red for dyn-act dyn-inact, respectively), the different shades represent the results obtained from three individual simulations.

We have observed similar fluctuations of the interdomain distance, both in the active and self-inactivated conformations (Figure 8(a)). For all of the examined trajectories, the values oscillated between 5–10 Å. The flexibility of the β-hairpins in the A’ conformers was similar in all structures which implies that there is no direct correlation between the loop mobility and the substitution of Asn to Ala in position 475.

**Figure 9.**
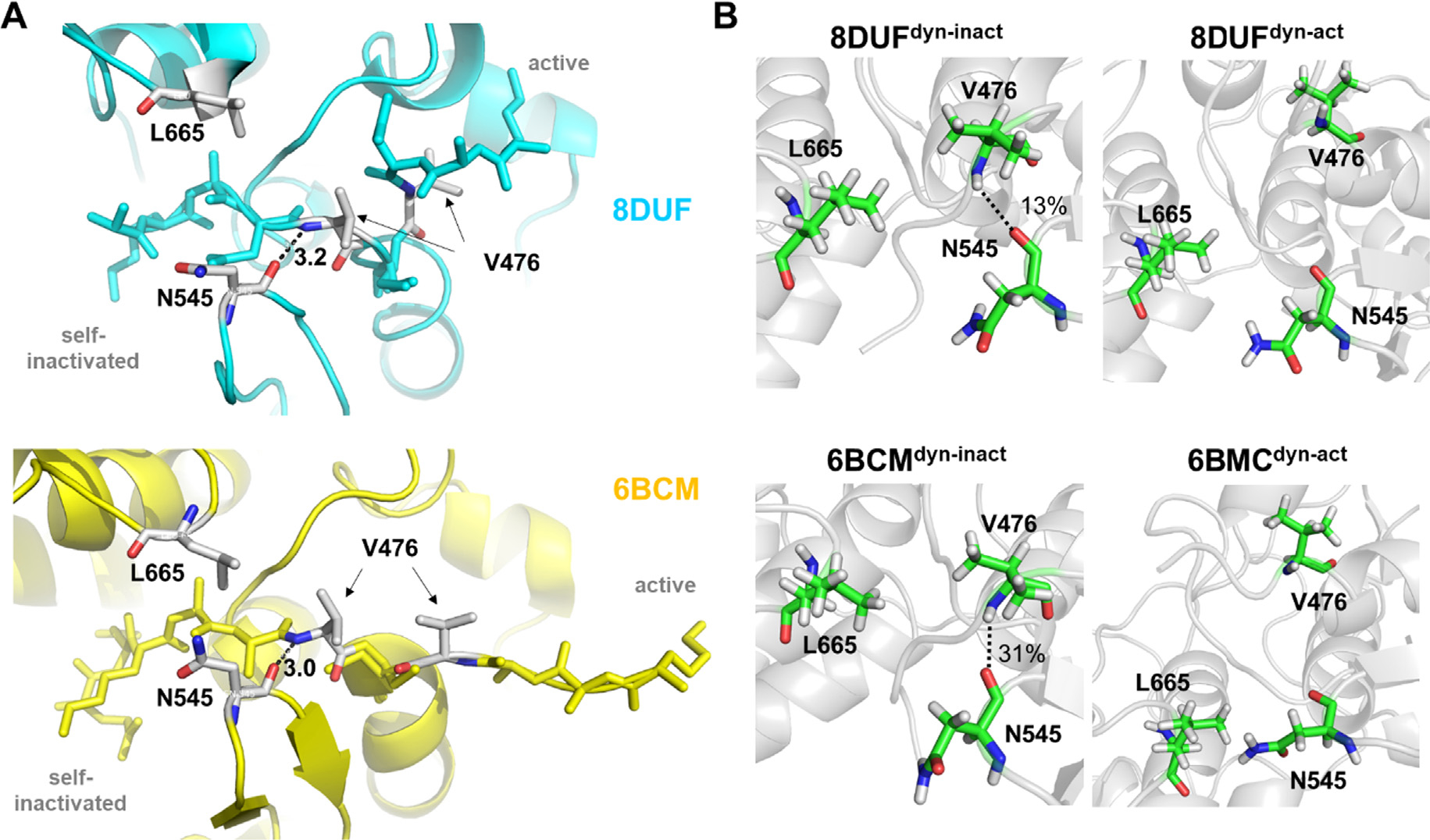
Interaction of N545 residue with the N terminus. **(a)** Distances (Å) between the main chain atoms of N545 and V476 residues are shown for wild-type (N475) and N475A mutant (A475) residue-containing enzymes based on crystal structures (PDB IDs: 8DUF and 6BCM, respectively). (**b**) The presence of H-bonds between the main chain atoms of N545 and V476 residues, the percentage values were determined based on the trajectories.

Movement of the β-hairpin loop has been observed previously in the N475A structure and the backbone-backbone contact between N545-O and V476-NH atoms may cause the relatively lower flexibility of the b-hairpin in the B’ conformer.^16^ Accordingly, we also observed that the main chain oxygen atom of N545 interacts with the main chain nitrogen atom of V476 in the self-inhibited conformation of the K741A/K767A mutant enzyme (PDB ID: 8DUF) (Figure 9(a)). Additionally, this is in agreement with the intramolecular contacts of the N terminus, which interacts during the trajectory with the residues of the β-hairpin (especially with N545) in the conformer B’, but not in A’.

Based on the MD analyses, the H-bond interaction between N545 and V476 residues was even more prevalent in the 6BCM^dyn-inact^ structure during the trajectories (Figure 9(b)). The distances between the V476 and N545 residues were found to show characteristic differences in the case of the structures containing a N475 (PDB ID: 8DUF and 2HWK) or A475 residue at the active site (PDB ID: 6BCM) (Figure 8(b)). The values for the A’ conformers (dyn-act) fluctuated between 10–15 Å, while the values for the B’ conformers were lower (between 5–8 Å), even for coordinates containing the wild-type (Asn) or the modified (Ala) residue in 475th position. The measured distances between the V476 and N545 residues showed considerably higher changes in case of the N475A mutant in its A’ conformer; the values were even comparable with those observed for the B’ conformer. In contrast, the distances determined for the active and self-inactivated conformations were not so comparable in the case of the enzyme containing N475 residue (8DUF^dyn-act^). This implied a higher structural flexibility as well as a less well-defined active conformation for the N475A mutant, indicating that the N475A mutation may make the enzyme more prone for adopting the B’ conformer.

### Key contacts at the active site in the active and self-inhibited conformations

In the crystal structure of the K741A/K767A mutant VEEV nsP2pro (PDB ID: 8DUF), the B’ conformer of the N terminus flips into the active site, facilitating the binding of ^472^AKANVC^477^ residues to the active site. This binding mode corresponds to the one observed for the N475A mutant (PDB ID: 6BCM) where the nucleophilic C477 residue occupies the S1′ site and residues 472–476 are bound to the S5-S1′ sites.^16^

To compare the self-inhibited conformations of the wild-type and N475A mutant enzymes, we mapped key contacts of the pseudo-substrate (intramolecular interactions) at the active site, and its binding mode was compared to that of an oligopeptide substrate (intermolecular interactions) (Table 3). The patterns of the interactions with the pseudo-substrates were found to be highly similar in almost all substrate-binding sites, proving the same binding mode of the N terminus to the active site in the self-inactivated conformation (8DUF^dyn-act^ and 6BCM^dyn-act^). We found only a limited number of hydrogen bonds between the enzyme and the substrate, which is in agreement with the finding of Russo et al. who identified mainly van der Waals interactions with the P4-P1′ residues.^23^ The binding mode of the N terminus was compared to that of an oligopeptide representing the wild-type nsP12 cleavage site of VEEV nsP2pro. Both ^472^-AKANVC^477^ residues of the N terminus and ^2^-QEAGAG^7^ residues of the oligopeptide substrate occupy the S5-S1′ sites. The comparison of the contact maps revealed that the interactions formed with the active site are highly similar in the case of the pseudo-substrate (N terminus) and the oligopeptide substrate at the S5-S1 sites, but alternative contacts are also formed in the self-inactivated conformations (Table 3).

**Figure 10.**
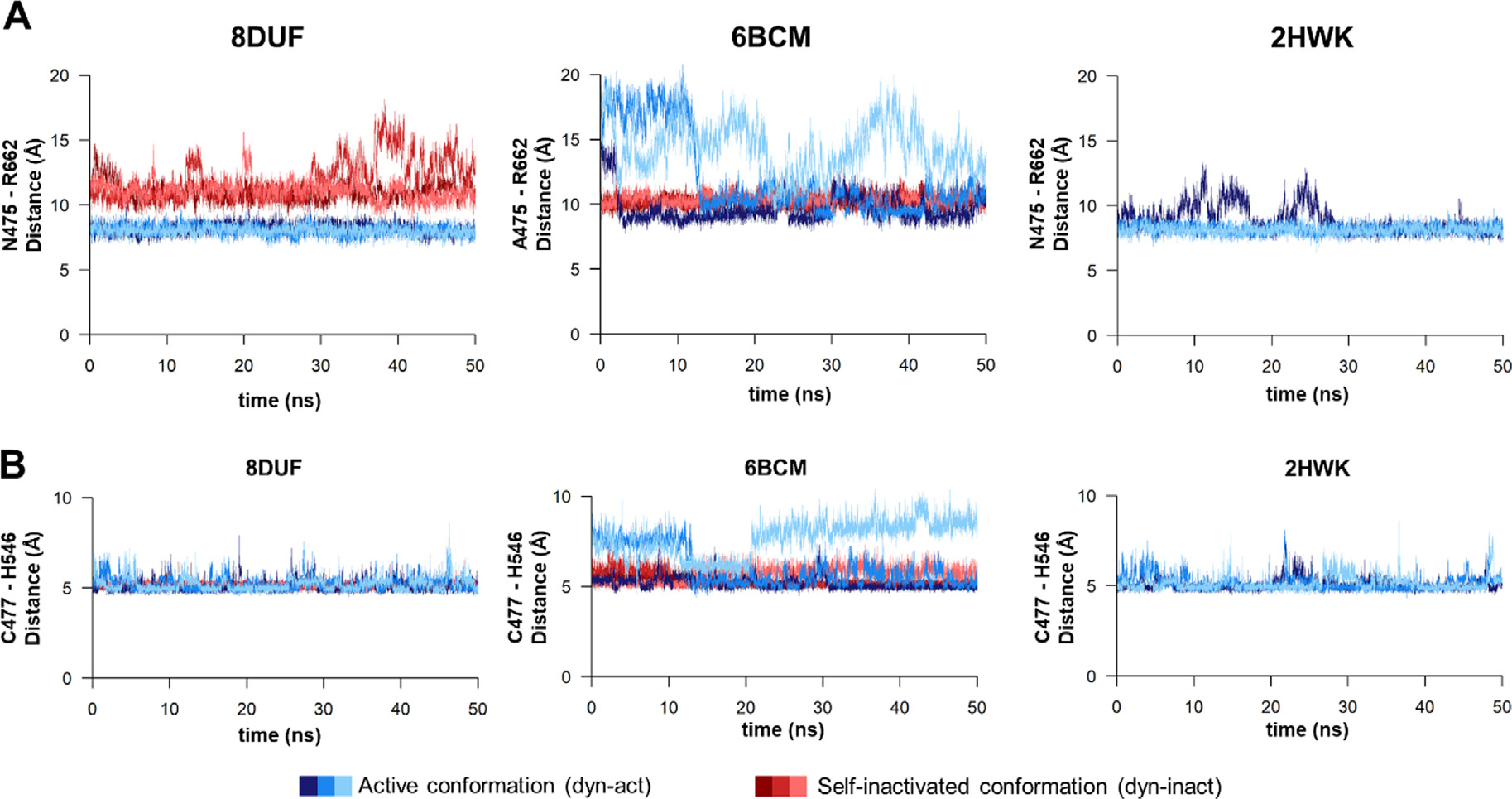
Distances between the N475-R662 and catalytic C477-H546 residues. **(a)** The distances (Å) between the center of mass of N475 and R662 residues during the trajectories. (**b**) The changes of the distance (Å) measured between the center of mass of the catalytic residues (C477 and H546) during the trajectories. Values are colored differentially for the A’ and B’ conformers (with blue and red for dyn-act dyn-inact, respectively), the different shades represent the results obtained from three individual simulations. The spatial positions of these residues at the active site are represented in Figure S4.

MD analysis of the wild-type VEEV nsP2pro complexed with the P34 substrate (RFDAGAYIFSSD, P6-P6′ residues) previously revealed that the substrate may exhibit significant conformational changes during the simulations, and the P1′-P6′ residues may be pointing not only towards the protease, but also towards the SAM MTase domain.^14^ We assumed that the conformational flexibility of the enzyme may interfere with the substrate binding, the binding of a substrate (or the N terminus) may be determined by the conformation and accessibility of the active site groove. In accordance with this, the interaction between the N475 and R662 residues was found previously to be important in the stabilization of the complex of SAM MTase and protease domains.^16–17^ Therefore, the contacts of residues 475 and 662 were analyzed in detail. The distance between these residues was comparable in the case of the N475-containing enzymes, but the A475-R622 distance (N475A mutant) became remarkably higher as compared to N475-R662 (wild-type) in the active conformations (Figure 10(a)). The crystal structure showed that the mutation of N475 to Ala does not alter the side chain conformation of the R662 residue,^6^ but our MD simulations revealed that the N475A mutation impairs interactions between the two domains, as we observed higher distances between the 475th and 662nd residues compared to the A’ conformers of the wild-type (N475) during the simulations. Our results demonstrate the importance of the interaction between the N475 and R662 residues in the stabilization of interdomain interactions. The distances between the C477 and the H546 catalytic residues showed moderate fluctuation and were more comparable in the A’ and B’ conformers. Interestingly, a simulation revealed even a 2-fold higher C477-H546 distance for the A’ conformer of N475A mutant enzyme (6BCM^dyn-act^) (Figure 10 (b)), indicating lower conformational stability for the catalytic site upon the mutation, the higher distance between the catalytic residues may potentially make the enzyme incapable for peptide bond hydrolysis.

The loss of these contacts between the SAM MTase and protease domains contribute to conformational changes that may potentially affect binding of the substrate, as well. This observation is in agreement with the increased distance between V476 and N545 residues (Figure 8(b)), because the increased distance between the protease and SAM MTase domains may potentially make nsP2pro more prone for the self-inactivated conformation, and the elevated structural flexibility is considered to affect substrate binding, as well. Our simulations predict a higher propensity for the N475A mutant to adopt the B’ conformer as compared to the enzymes lacking this mutation. In contrast to the N475A mutant, the K741A/K767A mutant (having the wild-type Asn residue in 475th position) showed larger fluctuations in its self-inactivated conformer ensembles, suggesting the role of conformational dynamics in the formation of the self-inactivated conformer.

## Conclusions

The Group IV viruses have a positive-sense single stranded RNA genome and are classified into six sub-classes (*Coronaviridae, Picornaviridae, Flaviviridae, Hepeviridae, Caliciviridae, Astroviridae,* and *Togaviridae*). The alphaviruses belong to *Togaviridae*. Multiple members of this family are emerging pathogens, including the VEEV and CHIKV viruses. The enzymes of the alphaviruses have been investigated for different reasons so far; i) nsP2 proteases of VEEV, SFV and SINV were studied as potential reagents for enzymatic removal of fusion tags from recombinant proteins *via* site-specific cleavage^25^; ii) multiple alphaviruses, such as VEEV, EEEV, WEEV, and CHIKV were considered to be agents for biological terrorism or biowarfare^4,26^; iii) the viral proteins are validated targets for antiviral drugs^27–29^; and iv) there is a need for efficient vaccination approaches.^30–31^ Neither virus-specific therapeutic approaches nor FDA-approved vaccines exist against the abovementioned viruses and their development is still challenging.^32^ Therefore, studies of enzymes encoded by alphaviruses, including the nsP2 proteases, may provide valuable information to understand their functional features as well as providing essential insights for structure-based drug design. In this paper, we describe the high-resolution crystal structure of K741A/K767A mutant VEEV nsP2pro containing wild-type N-terminal N475 residue and the molecular dynamics analysis of the wild-type (N475) and N475A mutant protein structures.

The structure of the K741A/K767A mutant VEEV nsP2pro has been determined by X-ray crystallography. This alphavirus nsP2pro structure has the highest resolution reported until October 2022 (1.46 Å) (Table 1). In addition to providing higher resolution data that may aid structure-based drug design, the structure of the K741A/K767A mutant VEEV nsP2pro was observed to exhibit two different conformations of the N-terminal loop just prior to the catalytic C477 residue. In the A’ conformer the active site of the enzyme (*i.e.* the cleft between the protease and SAM MTase domains) is opened because the N terminus is exposed to the protein surface, thus, the enzyme is competent for substrate binding. The B’ conformer represents an inactive conformation where the N terminus occupies the substrate binding site, mimicking substrate binding. The existence of the self-inactivated conformation is not unusual as it has been observed for the N475A mutant VEEV nsP2pro,^16^ but has not been previously reported for the wild-type (N475 residue-containing) enzyme. In this work we studied the structural characteristics of the wild-type and N475A mutant enzymes by comparing the free enzymes in the active and self-inactivated conformations, and the enzymes complexed with an oligopeptide substrate were also investigated.

As expected, a comparison of the wild-type (N475) VEEV nsP2pro structures revealed a close overlay of the N-terminal region in the A’ conformers, while the N475A mutant showed a different conformation, indicating a remarkable effect of the mutation on the conformation of the N terminus (Figure 3). The interaction maps of the N-terminal residues were similar in the self-inactivated conformations, indicating that the binding of the pseudo-substrate is mediated similarly in the wild-type (N475) and N475A mutant, as well. Although we did not observe N475A mutation-specific distribution of the interactions in the B’ conformers (Figure 7), an alternative pattern of interactions was determined for the pseudo- and the oligopeptide substrates (Table 3). Despite occupying the active site similarly, the interactions of the N terminus (enzyme) and peptide (substrate) residues with the substrate binding sites are more different.

The structural characteristics of the wild-type (N475) and N475A mutant enzymes were determined by comparing the structures before and after MD simulations. Our results imply that the self-inactivated conformation can be formed by the wild-type (N475) enzyme, as well, and not just the N475A mutation. Although the spatial orientation and intra-monomeric interactions of the pseudo-substrate are similar in the B’ conformer, the N terminus of the N475A mutant (PDB ID: 6BCM) does not overlay well with that of the K741A/K767A mutant enzyme (PDB ID: 8DUF) in the active conformation (A’ conformer). This indicates that the N475A mutation potentially makes the enzyme more prone to the formation of B’ conformer by changing the conformation of its N terminus (Figure 3) and by increasing the distance between protease and SAM MTase domains (Figure 8). Apparently, the N475A mutation impairs the key interdomain interactions (e.g. N475-R662), causing an increase of the distance between the N terminus and the SAM MTase domain and the flipping of the P6-P1 substrate residues out from the interdomain cleft (Figure 10). The longer distance between A475-R662 as compared to N475-R662 residues implies higher structural flexibility for the N475A mutant and the altered conformation of the active site is likely to be responsible for the substantial decrease of the catalytic efficiency observed *in vitro*.^14^ The substrate may be released from the active site more readily due to the weaker interdomain interactions.

Our results do not provide direct evidence about the molecular mechanisms behind the formation of the self-inactivated conformation, the possible factors and details of mechanisms that may trigger the inactivation of the enzyme remain to be identified. Based on our structural data, it cannot be determined unequivocally whether the mutations of the positively charged K741A and K767A SAM MTase residues increase the susceptibility of the protease domain to adopt a self-inactivated conformation or how allosteric properties of VEEV nsP2pro are changed. This is due to the fact that we have studied a truncated nsP2pro protein (residues 463–785) rather than the full-length nsP2 polyprotein or the nsP23 precleavage form, and the protease activity was not investigated *in vitro*. However, the possible effect of the surface mutations can be estimated based on the structural homology of SINV and VEEV nsP2 proteins. The R781 SINV nsp2 residue is located in the surface of SAM MTase domain, at the interface of the nsP2 and the linker connecting the macro and zinc-binding domains of nsP3. The R755 surface residue of SINV nsP2 is also surface-exposed, its side chain atoms may form hydrogen-bonds with D940 and T942 residues (PDB ID: 4GUA),^18^ thus it may mediate interactions between nsP2 and nsP3. Accordingly, we assume, that the K741 and K767 residues of VEEV nsP2pro may also contribute to the interactions of nsP2 and nsP3. The possible correlation of K741A/K767A mutations (or other SAM MTase surface sites) with the protease’s self-inactivation via yet unknown allosteric mechanisms may be of interest for *in silico* drug design. *In vitro*, the differentiation of the A’ and B’ conformer fractions may be necessary to properly titrate the enzyme and test how the buffer environment may influence enzyme inactivation. As the N475A mutant exhibited 4-fold higher K_M_ as compared to wild-type VEEV nsP2pro,^14^ the determination of the kinetic parameters may reveal whether the K741A/K767A mutations cause increase of K_M_ and are responsible for competitive inhibition *via* promoting binding of the N terminus to the active site. But, our study provides detailed information about the effects of N475A mutation which causes a decrease of catalytic efficiency *via* destabilization of the interactions between the SAM MTase and protease domains.

A limitation of our MD analysis is that it does not shed light on the dynamic properties of the full-length protein (1–794), because only the VEEV nsP2pro domain (473–768) was studied *in silico*. Nevertheless, it is important to note that in our study a protein encompassing R463-T785 residues of the VEEV nsp1234 polyprotein was crystallized. Consequently, the enzyme formed the self-inactivated conformation even though it had a longer N terminus (as compared to those residues that were visible in the electron density map: 473–785). Additionally, the effect of the N475A mutation was studied by investigating the VEEV nsP2pro containing residues 457–792 of the full-length VEEV nsP2,^16^ despite the fact that only the Q471-E791 residues were visible in the density map. The crystallized protein contained 18 residues upstream of residue 475, indicating that the inactive conformation was not necessarily formed due to the short length of the N terminus, but the dynamic properties of the N terminus may be different if more residues are present. Therefore, the movement of the N-terminal loop to the active site must be interpreted in the context of the truncated proteins. A more extensive analysis of the full-length proteins may reveal whether the autoinhibition of the wild-type enzyme is biologically relevant and contributes to substantial decrease of wild-type VEEV’s^15^ or SFV’s^19^ nsP2pro activity *in vitro*.

## Materials and Methods

### Cloning

An expression vector encoding surface entropy reduction mutations K741A and K767A was constructed in two steps using expression vector pDZ43, encoding hexahistidine (His_6_)-tagged maltose binding protein (MBP) from *Escherichia coli* followed in-frame by the Tobacco etch virus (TEV) protease recognition sequence and a truncated VEEV nsp2pro sequence (nsp1234 polyprotein R463 to T785), as starting material. pDZ43 was used as a template for QuikChange Lightning site-directed mutagenesis (Agilent, Santa Clara, CA) with primers PE2281 (5′-CATTG GGTACGATCGCGCGGCCCGTACGCACAAT-3′) and PE2282 (5′- ATTGTGCGTACGGGCCGCGCGATCGTACCCAATG-3′) to produce pDN2161, encoding His-MBP-TEV-VEEV K767A. Sequences were verified with sequencing primers PE29 (5′-GATGAAGCCCTGAAAGACGCGCAG-3′), PE30 (5′-GCAAGGCGATTAAGTTGGGTAACGC-3′), PE2285 (5′-ATGCGTGAGGTTCTTTGGAC-3′), PE2286 (5′-CAGCAGTGTGAAGACCATGC-3′), PE2287 (5′-AGGACAGTTCTGCCCTTCAA-3′), and PE2288 (5′-TTGGTCAAGGTTGATGAAAGC-3′). The single mutant pDN2161 was then used as a template for a second QuikChange reaction introducing the K741A mutation with primers PE2279 (5′-CTATAGCGCGGCAGTTCGCGTTTTCCCGGGTATGCA-3′) and PE2280 (5′-TGCATACCCGGGAAAACGCGAACTGCCGCGCTATAG-3′) to produce pDN2164, encoding His-MBP-TEV-VEEV protease K741A/K767A. Sequence changes were verified using PE29 and PE30.

### Protein expression and purification

pDN2164 was transformed into *E. coli* strain BL21-CodonPlus (DE3)-RIL (Agilent, Santa Clara, CA, USA). Cells were cultured at 37 °C in Luria broth with 100 μg ml^−1^ ampicillin, 30 μg ml^−1^ chloramphenicol and 0.2% glucose to mid-log phase. Production of the recombinant protease was induced with isopropyl β-D-1-thiogalactopyranoside (IPTG) at 1 mM, and the protein was expressed for four hours at 30 °C. The cells were pelleted by centrifugation and stored at −80 °C.

All protein purification procedures were conducted at 4 °C. Approximately 25 g of *E. coli* cell paste was re-suspended in 200 ml of ice-cold lysis buffer (50 mM phosphate buffered saline, pH 7.5, 150 mM sodium chloride, and 25 mM imidazole) supplemented with 5 protease inhibitor cocktail tablets (Roche Molecular Biochemicals). The cells were then passed three times through an APV-1000 homogenizer (Invensys APV Products, Albertslund, Denmark) at 69 MPa and centrifuged at 30,000 g for 30 minutes. The supernatant was collected and filtered through a 0.45 μm cellulose acetate membrane filter and applied onto a 20 ml HisPrep column (GE Healthcare, Piscataway, NJ, USA) that was pre-equilibrated with lysis buffer. The column was washed to baseline with lysis buffer and the protein was eluted from the column with a linear gradient from 30 to 250 mM imidazole using a buffer consisting of 50 mM phosphate buffered saline, 150 mM sodium chloride, and 250 mM imidazole. The fractions containing the protein were pooled together and concentrated with a YM30 membrane (EMD Millipore, Billerica, MA, USA). The concentrated sample was diluted 8x with 50 mM phosphate buffered saline, pH 7.5 and 150 mM sodium chloride and 5 mg of polyhistidine-tagged TEV protease was added to the solution and incubated overnight at 4 °C.^33^ The sample was then applied onto a 20 ml HisPrep column that was pre-equilibrated with 50 mM phosphate-buffered saline, 150 mM sodium chloride. The protein was isolated in the column flowthrough and was concentrated using an Amicon stirred cell with a YM10 membrane. The sample was clarified by centrifugation, filtered through a 0.22 mm membrane and applied onto a HiPrep 26/60 Sephacryl S-100 HR column that was pre-equilibrated with 25 mM Tris pH 7.5, 150 mM sodium chloride and 2 mM Tris(2-carboxyethyl) phosphine buffer. Peak fractions were pooled and concentrated to approximately 19.4 mg/ml (estimated at 280 nm using a molar extinction coefficient of 42860 M^−1^cm^−1^).^34^ Aliquots were flash-cooled with liquid nitrogen and stored at −80 °C. The molecular weight of 36553.7 Da was confirmed by electrospray ionization mass spectrometry (ESI-MS).

### Protein crystallization and X-ray diffraction data collection

Crystallization screens were set up using a Gryphon crystallization robot (Art Robbins, Sunnyvale, CA, USA) and commercially available crystallization screens. Sitting-drop vapor diffusion experiments were incubated at 18 °C. The best crystals used for data collection were obtained using the hanging-drop, vapor diffusion method by mixing 2 μl of protein (19.2 mg/ml) with 2 μl of well solution consisting of 2.0 M sodium formate and 10% (v/v) glycerol, sealing the drops over 500 ml of well solution (2.0 M sodium formate and 10% (v/v) glycerol), and incubating the plate at 18 °C. Plate-like crystals appeared within 1 week and were improved *via* streak seeding. For X-ray data collection, a single crystal was retrieved from the crystallization drop with a Litholoop and transferred to a 2 μl drop consisting of 2.0 M sodium formate and 20% (v/v) glycerol and soaked for 30 seconds. The crystal was retrieved with a Litholoop and immediately flash-cooled by rapidly plunging into liquid nitrogen.

X-ray diffraction data were collected remotely on the SER-CAT beamline 22-ID of the Advanced Photon Source, Argonne National Laboratory using a MAR300 CCD detector. A data set from a single crystal held at −180 °C was obtained using an X-ray wavelength of 1.00000 Å, a crystal to detector distance of 150 mm, an X-ray exposure time of 3 seconds and a 0.5° oscillation angle. A total of 360 frames of data were collected. The diffraction data were processed using HKL-3000.^35^ The data collection and refinement statistics are presented in the Results section, Table 2). Initial phases were obtained by molecular replacement using the program Phaser in the Phenix suite of programs.^36^ The coordinates from the PDB deposition 2HWK (VEEV nsp2 protease domain) were used as a search model after removing all nonprotein atoms.^13^ Model rebuilding and refinements were conducted with Coot^37^ and phenix.refine.^38^ The occupancies of the N terminus (%) were determined by setting the initial occupancy value for both A’ and B’ conformers at 0.5 and then running occupancy refinement in phenix.refine. Water molecules were located with Coot, manually inspected, and refined with phenix.refine. Model validation was performed with MolProbity.^39^ The coordinates and structure-factor files were deposited into the Protein Data Bank with accession code 8DUF.

### Coordinate files and molecular dynamics (MD) simulations

The coordinate files of VEEV nsP2pro were downloaded from the PDB^40^ and used to study its active and self-inactivated conformations by analysis of the free enzymes and enzyme-substrate complexes by MD simulations (Table 4).

The 8DUF, 6BCM, and 2HWK coordinate files were used as the starting structures for the simulations. The structures were aligned to 6BCM using the Chimera software.^41^ Besides the proteins and the water molecules, every other moiety was removed from the crystal structures. Chains were treated as continuous protein structures, any assignments denoting termination were ignored. In case of 8DUF, residues up until A768 were considered. The active and self-inactivated conformers were investigated separately. Protonation state of His residues were determined with Chimera. Propka3 was used to determine the protonation state of titratable residues.^42–43^ N-terminal acetyl and C-terminal N-methylamide groups were added with GaussView 6.^44^ Missing hydrogens were automatically added with the tleap module of AmberTools16. The MD simulation protocols were based on the method described previously.^15^ The catalytic residues were considered to be in their activated forms, i.e. the Cys was deprotonated and the His was protonated. The studied VEEV nsP2pro structures are listed in Table 4.

The simulations were carried out with the Amber16^45^ software, applying GPU acceleration.^46–47^ Parameters for the proteins and the substrates were taken from the FF14SB^48^ force field. The systems were immersed in water. The TIP3P^49^ model was used to describe the water molecules. Sodium and chloride ions were added to approximate the salt concentration of 0.15 mol $\left(c_{\text{ion}}\right)$, applying the addionsrand command in tleap to replace individual water molecules. To calculate the number of ions, we applied the following equations.


(1) $$N^{\text{avg}}=\frac{N_{\text{wat}}*C_{\text{ion}}}{55.5}$$

The average number of ions was calculated based on the number of water molecules present in the simulation box ($N_{\text{wat}}$) and the macroscopic salt concentration, 0.15 mol $\left(c_{ion}\right)$. To calculate the number of monovalent cations and anions, we modified the average ion number according to the charge of the protein system, as described in the following equations.


(2) $$N^{+}=N^{avg}-\frac{Q}{2}$$

(3) $$N^{-}=N^{avg}+\frac{Q}{2}$$

In the equations, Q represents the charge of the protein system, while $N^{+}$ and $N^{-}$ describe the number of cations and anions, respectively. This process is based on the method described in literature.^50^

The systems were first minimized with the steepest descent method for a duration of 100,000 steps, followed by further optimization with the conjugate gradient method for 25,000 steps. Each system was heated to 300 K during a 1 ns long simulation in an NVT ensemble, followed by a 0.5 ns long NVT equilibration at 300 K. During the whole course of the simulations, Langevin dynamics temperature regulation was applied, and SHAKE^51^ was performed for bonds involving hydrogen atoms. A timestep of 1 fs was applied. The cut-off for the non-bonded interactions was 10 Å. To describe long-range electrostatic interactions, the Particle Mesh Ewald (PME) method was applied.^52^ Positional restraints with force constants of 10 kcal mol^−1^ Å^−2^ were applied for the protein atoms. During the whole course of the simulations, Langevin dynamics temperature regulation was applied, SHAKE was performed for bonds involving hydrogen atoms, and a timestep of 1 fs was applied. The NVT equilibration was followed by a 0.5 ns equilibration on 300 K in an NPT ensemble. Following the equilibration, the systems were heated to 400 K during a 0.5 ns long simulation, and a 1 ns long equilibration was carried out at 400 K. The systems were cooled to 5 K during a 2.5 ns long simulation. This was followed by another cycle of heating to 400 K, equilibration and cooling back to 5 K, applying positional restraints with force constants of 5 kcal mol^−1^ Å^−2^ on protein atoms. This was followed by two further cycles, applying harmonic restraints with a force constant of 3 kcal mol^−1^ Å^−2^ and 1 kcal mol^−1^ Å^−2^ on the backbone atoms of the protein, respectively. Finally, without any positional constraints, the systems were equilibrated at 5 K for 1.5 ns, heated to 300 K during 1.5 ns, and equilibrated for 5 ns at 300 K. The production runs were carried out at 300 K, for 50 ns. Three individual simulations were carried out for each of the examined protein structures, with a sampling time of 5 ps. To calculate the root-mean-square deviation (r.m.s.d.), the cpptraj^53^ program was applied; both main chain and side chain atoms were included while calculating r.m.s.d. values and the reference structure was calculated as the average of every 10th frame of all three production runs. The ensembles were clustered with cpptraj.

### Accession Numbers

The atomic coordinates have been deposited in the Protein Data Bank with ID 8DUF.

## Supplementary Material

Supplementary

## Figures and Tables

**Table 1:** Coordinate files of the VEEV, CHIKV and Sindbis virus proteases in Protein Data Bank. The database was last assessed on September 1, 2022. E64d is a peptide-like epoxysuccinyl inhibitor. Mutations are shown based on numbering of nsP2 and not polyprotein (the polyprotein sequences are represented in Figure S1).

Coordinate file	Virus	Method	Resolution	Conformation	Mutation	Ligand	Reference

8DUF.pdb	VEEV	X-ray	1.46 Å	active + inactive	K741A, K767A	no	This study
6BCM.pdb	VEEV	X-ray	2.10 Å	active + inactive	N475A	no	^16^
2HWK.pdb	VEEV	X-ray	2.45 Å	active	no	no	^13^
5EZS.pdb	VEEV	X-ray	2.16 Å	active	no	E64d	^14^
5EZQ.pdb	VEEV	X-ray	1.66 Å	active	no	no	^14^
3TRK.pdb	CHIKV	X-ray	2.40 Å	active	no	no	not available
4ZTB.pdb	CHIKV	X-ray	2.59 Å	active	no	no	^17^
4GUA.pdb	SINV	X-ray	2.85 Å	active	no	no	^18^

**Table 2:** X-ray crystallography data collection and refinement statistics for VEEV nsP2pro.

Diffraction source	SER-CAT, 22-ID
Wavelength (Å)	1.00000
Temperature (K)	100
Detector	MAR300 CCD
Space group	*P*2_1_2_1_2_1_
***Unit cell parameters***	
a,b,c (Å)	45.19, 46.17, 175.95
α = β = γ (°)	90
Resolution range (Å)	50–0.1.46 (1.49–1.46)
Total reflections	376,179
Unique reflections	64,078 (3040)
Completeness (%)	98.6 (95.8)
Multiplicity	5.9 (5.3)
Mean *l*/σ(*l*)	31.7 (2.0)
R_merge_	0.090 (1.53)
R_p.i.m._	0.039 (0.662)
CC_1/2_	0.994 (0.545)
***Refinement Statistics***	
Resolution range (Å)	40.19–1.46
Number of reflections	63,967
Number of reflections used in *R*_free_	3182
Final *R*_work_	0.154 (0.190)
Final *R*_free_	0.197 (0.294)
***Number of non-H atoms***	
Protein	2579
Water	326
**Average *B* factors (Å^2^)**	
Protein	21.5
Water	37.9
Estimated coordinate error (Å)	0.13
***R.m.s. deviations from ideal***	
Bond lengths (Å)	0.005
Bond angles (°)	0.7
**Ramachandran plot**	
Favored (%)	98.7
Allowed (%)	1.3
Outliers (%)	-

**Table 3:** Interactions of the VEEV nsP2pro active site residues with a peptide substrate and the N terminus of the protease domain. The interactions at the active site are shown for the binding of the residues of an oligopeptide substrate or the N terminus (“dyn-inact” structures). The hydrophobic contacts and the H-bonds are also included in the table. The residues of the binding sites forming H-bonds with those of the substrate are underlined. The values in parentheses indicate the number of observed H-bonds. The hydrophobic interactions were mapped based on a modeled enzyme-substrate complex and on the cluster representative structure for the self-inactivated conformations extracted from the simulations. The LigPlot+^24^ software was used to map the hydrophobic interactions.

Binding site residues *	Substrate residue	Enzyme-substrate complex **	N-terminal residue (8DUF / 6BCM)	8DUF_dyn-inact_	6BCM_dyn-inact_

-	**P6-Leu1**	S511, E513, I542	**- / Q471**	-	-
-	**P5-Gln2**	I514, I698, S701, A728, I732	**A472 / N472**	M702	I514, I698, S701, A728, S731, I732
S511, E513, W547, M702, K706*	**P4-Glu3**	H510, S511, W547, I698, S701, M702, K706	**K473 / K473**	I542, W547	S511, I542, W547
H510, I542, W547, I698, M702	**P3-Ala4**	A509, H510, I542, D664, I698, M702	**A474 / A474**	A509, H510, D664, I698, M702	A509, H510, D664, M701
W478, C477, A509, H510, W547	**P2-Gly5**	W478, A509, H510, N545, W547	**N475 / A475**	A472, K473, A509,	A509, H510 (2), I542, N545, H546, W547
				H510 (2), I542, N545, W547	
N475, C477, W478, A509, N545, H546, L665	**P1-Ala6**	C477, W478, N545, H546, W547	**V476 / V476**	N545, H546, W547	N545, H546, W547
A474, N475, C477, K480, H546	**P1'-Gly7**	N475, C477,	C477 / C477	A479, K480, A481, H546	A479, K480, A481, H546, W547
		R662 (2)	**W478 / W478**	K480, L482, F504, K508, H510, W547, V615	A479, K480, A481, L482, V501, D502, Y503, F504, K508

* The substrate binding site-forming residues have been determined previously based on the complex of VEEV nsP2pro and a substrate representing P4-P1' residues of nsP12 cleavage site (EAGAG).^23^

** The enzyme-substrate interactions were mapped based on a modeled complex of VEEV nsP2 protease and LQEAGAjGSVETP substrate.^14^ The coordinate file was kindly provided previously^15^ by Patricia M. Legler (Center for Bio/molecular Science and Engineering, U.S. Naval Research Laboratory).

**Table 4:** Studied VEEV nsP2pro structures. The table shows the names of the structures that were prepared based on the indicated templates and were used for MD simulations. We studied the standalone enzymes having active (A’ conformer, “dyn-act”) or self-inactivated conformation (B’ conformer, “dyn-inact”). The wild-type and mutant residues of the SAM MTase and protease domains are also shown.

Structure name	Template PDB	Conformer/Conformation	Substrate	SAM MTase / Protease

8DUF^dyn-act^	8DUF	A’ / active	no	(K741A/K767A) / N475
8DUF^dyn-mact^	8DUF	B’ / self-inactivated	no	(K741A/K767A) / N475
6BCM^dyn-act^	6BCM	A’ / active	no	(K741/K767) / N475A
6BCM^dyn-inact^	6BCM	B’ / self-inactivated	no	(K741/K767) / N475A
2HWK^dyn-act^	2HWK	A’ / active	no	(K741/K767) / N475
