# Supplementary material for: Self-inhibited State of Venezuelan Equine Encephalitis Virus (VEEV) nsP2 Cysteine Protease: A Crystallographic and Molecular Dynamics Analysis
Source: J Mol Biol. Author manuscript; Available in PMC 2024 Jan 1. (PMC10758287; doi:10.1016/j.jmb.2023.168012)

Figure S1. Sequence alignment of polyprotein sequences. Sequences of Venezuelan equine encephalitis virus (VEEV), Sindbis virus (SINV) and Chikungunya virus (CHIK) polyproteins were aligned using Align module of UniProt database. UniProt identifiers are shown for all sequences. Sequences are numbered according to polyprotein numbering, but numbering for VEEV nsP2 is also shown (VEEV_nsP2). The region of nsP2 that is included in the structural file (PDB ID: 8DUF) is highlighted by green background. The VEEV nsP2 encompasses 536-1329 residues. The nsP1*2, nsP2*3 and nsP3*4 cleavage sites sequences (P5-P5' residues) are highlighted by cyan background in the sequence of VEEV polyprotein. The crystallized protein contained alanine residues in K741 and K767 positions, the wild-type residues are bold and red in the sequence of VEEV nsP2pro.

```
VEEV_Q9WIJ1   --MEKVHVDIEEDSPFLRALQRSFPQFEVEAKQVTDNDHANARAFSHLASKLIETEVDPS      58
VEEV_P27282   --MEKVHVDIEEDSPFLRALQRSFPQFEVEAKQVTDNDHANARAFSHLASKLIETEVDPS      58
SINV_P03317   MEKPVVNVDVDPQSPFVVQLQKSFPQFEVVAQQVTPNDHANARAFSHLASKLIELEVPTT      60
CHIK_Q8JUX6   --MDPVYVDIDADSAFLKALQRAYPMFEVEPRQVTPNDHANARAFSHLAIKLIEQEIDPD      58
CHIK_A6MH22   --MDPVYVDIDADSAFLKALQRAYPMFEVEPRQVTPNDHANARAFSHLAIKLIEQEIDPD      58
                   * **:: :* *:  **:::* ***  :*** ************* **** *:

VEEV_Q9WIJ1   DTILDIGSAPARRMYSKHKYHCICPMRCAEDPDRLYKYATKLKKNCKEITDKELDKKMKE      118
VEEV_P27282   DTILDIGSAPARRMYSKHKYHCICPMRCAEDPDRLYKYATKLKKNCKEITDKELDKKMKE      118
SINV_P03317   ATILDIGSAPARRMFSEHQYHCVCPMRSPEDPDRMMKYASKLAEKACKITNKNLHEKIKD      120
CHIK_Q8JUX6   STILDIGSAPARRMMSDRKYHCVCPMRSAEDPERLANYARKLASAAGKVLDRNISGKIGD      118
CHIK_A6MH22   STILDIGSAPARRMMSDRKYHCVCPMRSAEDPERLANYARKLASAAGKVLDRNISGKIGD      118
               ************* *.::***:****. ***:*: :** ** . . :: ::::  *: :

VEEV_Q9WIJ1   LAAVMSDPDLETETMCLHDDESCRYEGQVAVYQDVYAVDGPTSLYHQANKGVRVAYWIGF      178
VEEV_P27282   LAAVMSDPDLETETMCLHDDESCRYEGQVAVYQDVYAVDGPTSLYHQANKGVRVAYWIGF      178
SINV_P03317   LRTVLDTPDAETPSLCFHNDVTCNMRAEYSVMQDVYI-NAPGTIYHQAMKGVRTLYWIGF      179
CHIK_Q8JUX6   LQAVMAVPDTETPTFCLHTDVSCRQRADVAIYQDVYAVHAPTSLYHQAIKGVRLAYWVGF      178
CHIK_A6MH22   LQAVMAVPDTETPTFCLHTDVSCRQRADVAIYQDVYAVHAPTSLYHQAIKGVRVAYWVGF      178
              * :*:  ** ** ::*:* * :*. ..: :: ****  ..* ::**** ****  **:**

VEEV_Q9WIJ1   DTTPFMFKNLAGAYPSYSTNWADETVLTARNIGLCSSDVMERSRRGMSILRKKYLKPSNN      238
VEEV_P27282   DTTPFMFKNLAGAYPSYSTNWADETVLTARNIGLCSSDVMERSRRGMSILRKKYLKPSNN      238
SINV_P03317   DTTQFMFSAMAGSYPAYNTNWADEKVLEARNIGLCSTKLSEGRTGKLSIMRKKELKPGSR      239
CHIK_Q8JUX6   DTTPFMYNAMAGAYPSYSTNWADEQVLKAKNIGLCSTDLTEGRRGKLSIMRGKKLEPCDR      238
CHIK_A6MH22   DTTPFMYNAMAGAYPSYSTNWADEQVLKAKNIGLCSTDLTEGRRGKLSIMRGKKLKPCDR      238
              *** **:. :**:**:*.****** ** *:******:.: *     :**:* * *:* ..

VEEV_Q9WIJ1   VLFSVGSTIYHEKRDLLRSWHLPSVFHLRGKQNYTCRCETIVSCDGYVVKRIAISPGLYG      298
VEEV_P27282   VLFSVGSTIYHEKRDLLRSWHLPSVFHLRGKQNYTCRCETIVSCDGYVVKRIAISPGLYG      298
SINV_P03317   VYFSVGSTLYPEHRASLQSWHLPSVFHLNGKQSYTCRCDTVVSCEGYVVKKITISPGITG      299
CHIK_Q8JUX6   VLFSVGSTLYPESRKLLKSWHLPSVFHLKGKLSFTCRCDTVVSCEGYVVKRITMSPGLYG      298
CHIK_A6MH22   VLFSVGSTLYPESRKLLKSWHLPSVFHLKGKLSFTCRCDTVVSCEGYVVKRITMSPGLYG      298
              * ******:* * *  *:**********.** .:****:*:***:*****:*::***: *

VEEV_Q9WIJ1   KPSGYAATMHREGFLCCKVTDTLNGERVSFPVCTYVPATLCDQMTGILATDVSADDAQKL      358
VEEV_P27282   KPSGYAATMHREGFLCCKVTDTLNGERVSFPVCTYVPATLCDQMTGILATDVSADDAQKL      358
SINV_P03317   ETVGYAVTHNSEGFLLCKVTDTVKGERVSFPVCTYIPATICDQMTGIMATDISPDDAQKL      359
CHIK_Q8JUX6   KTTGYAVTHHADGFLMCKTTDTVDGERVSFSVCTYVPATICDQMTGILATEVTPEDAQKL      358
CHIK_A6MH22   KTTGYAVTHHADGFLMCKTTDTVDGERMSFSVCTYVPATICDQMTGILATEVTPEDAQKL      358
              :  ***.* : :*** **.***:.***:** ****:***:*******:**::: :*****

VEEV_Q9WIJ1   LVGLNQRIVVNGRTQRNTNTMKNYLLPVVAQAFARWAKEYKEDQEDERPLGLRDRQLVMG      418
VEEV_P27282   LVGLNQRIVVNGRTQRNTNTMKNYLLPVVAQAFARWAKEYKEDQEDERPLGLRDRQLVMG      418
SINV_P03317   LVGLNQRIVINGRTNRNTNTMQNYLLPIIAQGFSKWAKERKDDLDNEKMLGTRERKLTYG      419
CHIK_Q8JUX6   LVGLNQRIVVNGRTQRNTNTMKNYMIPVVAQAFSKWAKECRKDMEDEKLLGVRERTLTCC      418
CHIK_A6MH22   LVGLNQRIVVNGRTQRNTNTMKNYLLPVVAQAFSKWAKECRKDMEDEKLLGVRERTLTCC      418
              *********:****:******:**::*::**.*::**** :.* ::*: ** *:* *.

VEEV_Q9WIJ1   CCWAFRRHKITSIYKRPDTQTIIKVNSDFHSFVLPRIGSNTLEIGLRTRIRKMLEEHKEP      478
VEEV_P27282   CCWAFRRHKITSIYKRPDTQTIIKVNSDFHSFVLPRIGSNTLEIGLRTRIRKMLEEHKEP      478
SINV_P03317   CLWAFRTKKVHSFYRPPGTQTCVKVPASFSAFPMSSVWTTSLPMSLRQKLKLALQPKKEE      479
CHIK_Q8JUX6   CLWAFKKQKTHTVYKRPDTQSIQKVQAEFDSFVVPSLWSSGLSIPLRTRIKWLLSKVPKT      478
CHIK_A6MH22   CLWAFKKQKTHTVYKRPDTQSIQKVQAEFDSFVVPSLWSSGLSIPLRTRIKWLLSKVPKT      478
              * ***: :*  :.*: *.**:  ** :.* :* :  : :. * : ** :::  *.   :
```

```
VEEV_Q9WIJ1    SPLITAE-DVQEAKCAADEAKEVREAEELRAALPPLAADVEE---PTLEADVDLMLQEAG     534
VEEV_P27282    SPLITAE-DVQEAKCAADEAKEVREAEELRAALPPLAADVEE---PTLEADVDLMLQEAG     534
SINV_P03317    KLLQVSEELVMEAKAAFEDAQEEARAEKLREALPPLVADKGIEAAAEVVCEVEGLQADIG     539
CHIK_Q8JUX6    DLTPYSG-DAQEARDAEKEAEEEREAELTLEALPPLQAAQED---VQVEIDVEQLEDRAG     534
CHIK_A6MH22    DLIPYSG-DAQEARDAEKEAEEEREAELTREALPPLQAAQED---VQVEIDVEQLEDRAG     534
               .    :   . **: * .:*:*  .**    ***** *          :  :*: :    *

               nsP1*2
VEEV_nsP2       1.........10........20........30........40........50.......59
VEEV_Q9WIJ1    AGSVETPRGLIKVTSYAGEDKIGSYAVLSPQAVLKSEKLSCIHPLAEQVIVITHSGRKGR     594
VEEV_P27282    AGSVETPRGLIKVTSYAGEDKIGSYAVLSPQAVLKSEKLSCIHPLAEQVIVITHSGRKGR     594
SINV_P03317    AALVETPRGHVRIIPQANDRMIGQYIVVSPNSVLKNAKLAPAHPLADQVKIITHSGRSGR     599
CHIK_Q8JUX6    AGIIETPRGAIKVTAQPTDHVVGEYLVLSPQTVLRSQKLSLIHALAEQVKTCTHSGRAGR     594
CHIK_A6MH22    AGIIETPRGAIKVTAQPTDHVVGEYLVLSPQTVLRSQKLSLIHALAEQVKTCTHSGRAGR     594
               *. :****** :::      :   :*.* *:**::**:. **:  * **:**    ***** **

VEEV_nsP2      60........70........80........90........100.......110......119
VEEV_Q9WIJ1    YAVEPYHGKVVVPEGHAIPVQDFQALSESATIVYNEREFVNRYLHHIATHGGALNTDEEY     654
VEEV_P27282    YAVEPYHGKVVVPEGHAIPVQDFQALSESATIVYNEREFVNRYLHHIATHGGALNTDEEY     654
SINV_P03317    YAVEPYDAKVLMPAGGAVPWPEFLALSESATLVYNEREFVNRKLYHIAMHGPAKNTEEEQ     659
CHIK_Q8JUX6    YAVEAYDGRVLVPSGYAISPEDFQSLSESATMVYNEREFVNRKLHHIAMHGPALNTDEES     654
CHIK_A6MH22    YAVEAYDGRVLVPSGYAISPEDFQSLSESATMVYNEREFVNRKLHHIAMHGPALNTDEES     654
               **** *..:*::* * *:    :* :******:********** *:*** ** * **:**

VEEV_nsP2      120.......130.......140.......150.......160.......170......179
VEEV_Q9WIJ1    YKTVKPSEHDGEYLYDIDRKQCVKKELVTGLGLTGELVDPPFHEFAYESLRTRPAAPYQV     714
VEEV_P27282    YKTVKPSEHDGEYLYDIDRKQCVKKELVTGLGLTGELVDPPFHEFAYESLRTRPAAPYQV     714
SINV_P03317    YKVTKAELAETEYVFDVDKKRCVKKEEASGLVLSGELTNPPYHELALEGLKTRPAVPYKV     719
CHIK_Q8JUX6    YELVRAERTEHEYVYDVDQRRCCKKEEAAGLVLVGDLTNPPYHEFAYEGLKIRPACPYKI     714
CHIK_A6MH22    YELVRAERTEHEYVYDVDQRRCCKKEEAAGLVLVGDLTNPPYHEFAYEGLKIRPACPYKI     714
               *:  .:  .   : **::*:*:::* *** .:** * *:*.:**:**:* *.*: *** **::

VEEV_nsP2      180.......190.......200.......210.......220.......230......239
SINV_P03317    ETIGVIGTPGSGKSAIIKSTVTARDLVTSGKKENCREIEADVLRLRGMQITSKTVDSVML     779
VEEV_Q9WIJ1    PTIGVYGVPGSGKSGIIKSAVTKKDLVVSAKKENCAEIIRDVKKMKGLDVNARTVDSVLL     774
VEEV_P27282    PTIGVYGVPGSGKSGIIKSAVTKKDLVVSAKKENCAEIIRDVKKMKGLDVNARTVDSVLL     774
CHIK_Q8JUX6    AVIGVFGVPGSGKSAIIKNLVTRQDLVTSGKKENCQEITTDVMRQRGLEISARTVDSLLL     774
CHIK_A6MH22    AVIGVFGVPGSGKSAIIKNLVTRQDLVTSGKKENCQEITTDVMRQRGLEISARTVDSLLL     774
                 .*** *.******.***. ** :***.*.***** **   ** : :*:::.::****::*

VEEV_nsP2      240.......250.......260.......270. ......280.......290....297
VEEV_Q9WIJ1    NGCKHPVETLYIDEAFACHAGTLRALIAIIRPKK-AVLCGDPKQCGFFNMMCLKVHFNH-     832
VEEV_P27282    NGCKHPVETLYIDEAFACHAGTLRALIAIIRPKK-AVLCGDPKQCGFFNMMCLKVHFNH-     832
SINV_P03317    NGCHKAVEVLYVDEAFACHAGALLALIAIVRPRKKVVLCGDPMQCGFFNMMQLKVHFNHP     839
CHIK_Q8JUX6    NGCNRPVDVLYVDEAFACHSGTLLALIALVRPRQKVVLCGDPKQCGFFNMMQMKVNYNH-     833
CHIK_A6MH22    NGCNRPVDVLYVDEAFACHSGTLLALIALVRPRQKVVLCGDPKQCGFFNMMQMKVNYNH-     833
               ***:: *:.**:*******:*:* ****::**:: .****** ******** :**::**

VEEV_nsP2        298.........310.......320.......330.......340.......350..355
VEEV_Q9WIJ1    --EICTQVFHKSISRRCTKSVTSVVSTLFYDKKMRTTNPKETKIVIDTTGSTKPKQDDLI     890
VEEV_P27282    --EICTQVFHKSISRRCTKSVTSVVSTLFYDKKMRTTNPKETKIVIDTTGSTKPKQDDLI     890
SINV_P03317    EKDICTKTFYKYISRRCTQPVTAIVSTLHYDGKMKTTNPCKKNIEIDITGATKPKPGDII     899
CHIK_Q8JUX6    --NICTQVYHKSISRRCTLPVTAIVSSLHYEGKMRTTNEYNKPIVVDTTGSTKPDPGDLV     891
CHIK_A6MH22    --NICTQVYHKSISRRCTLPVTAIVSSLHYEGKMRTTNEYNKPIVVDTTGSTKPDPGDLV     891
                 :***:.::* ******  **::**:*.*: **:***    :. * :* **:***. .*::

VEEV_nsP2      356.360.......370.......380.......390.......400.......410..415
VEEV_Q9WIJ1    LTCFRGWVKQLQIDYKGNEIMTAAASQGLTRKGVYAVRYKVNENPLYAPTSEHVNVLLTR     950
VEEV_P27282    LTCFRGWVKQLQIDYKGNEIMTAAASQGLTRKGVYAVRYKVNENPLYAPTSEHVNVLLTR     950
SINV_P03317    LTCFRGWVKQLQIDYPGHEVMTAAASQGLTRKGVYAVRQKVNENPLYAITSEHVNVLLTR     959
CHIK_Q8JUX6    LTCFRGWVKQLQIDYRGHEVMTAAASQGLTRKGVYAVRQKVNENPLYASTSEHVNVLLTR     951
CHIK_A6MH22    LTCFRGWVKQLQIDYRGYEVMTAAASQGLTRKGVYAVRQKVNENPLYASTSEHVNVLLTR     951
               *************** * *:****************** ********* **********
```

```
VEEV_nsP2      416.420.......430.......440.......450.......460.......470..475
VEEV_Q9WIJ1    TEDRIVWKTLAGDPWIKTLTAKYPGNFTATIEEWQAEHDAIMRHILERPDPTDVFQNKAN     1010
VEEV_P27282    TEDRIVWKTLAGDPWIKTLTAKYPGNFTATIEEWQAEHDAIMRHILERPDPTDVFQNKAN     1010
SINV_P03317    TEDRLVWKTLQGDPWIKQPTNIPKGNFQATIEDWEAEHKGIIAAINSPTPRANPFSCKTN     1019
CHIK_Q8JUX6    TEGKLVWKTLSGDPWIKTLQNPPKGNFKATIKEWEVEHASIMAGICSHQMTFDTFQNKAN     1011
CHIK_A6MH22    TEGKLVWKTLSGDPWIKTLQNPPKGNFKATIKEWEVEHASIMAGICSHQMTFDTFQNKAN     1011
               **.::***** ******       *** ***::*:.** .*:  * .     : *. *:*

VEEV_nsP2      476.480.......490.......500 .......510.......520.......530.534
VEEV_Q9WIJ1    VCWAKALVPVLKTAGIDMTTEQWNTV-DYFETDKAHSAEIVLNQLCVRFFGLDLDSGLFS     1069
VEEV_P27282    VCWAKALVPVLKTAGIDMTTEQWNTV-DYFETDKAHSAEIVLNQLCVRFFGLDLDSGLFS     1069
SINV_P03317    VCWAKALEPILATAGIVLTGCQWSELFPQFADDKPHSAIYALDVICIKFFGMDLTSGLFS     1079
CHIK_Q8JUX6    VCWAKSLVPILETAGIKLNDRQWSQIIQAFKEDKAYSPEVALNEICTRMYGVDLDSGLFS     1071
CHIK_A6MH22    VCWAKSLVPILETAGIKLNDRQWSQIIQAFKEDKAYSPEVALNEICTRMYGVDLDSGLFS     1071
               *****:* *:* **** :.  **. :    *  ** :*   .*: :* :::*:** *****

VEEV_nsP2      535..540.        ......550.......560.......570.......580....587
VEEV_Q9WIJ1    APTVPLSIR-------NNHWDNSPSPNMYGLNKEVVRQLSRRYPQLPRAVATGRVYDMNT     1122
VEEV_P27282    APTVPLSIR-------NNHWDNSPSPNMYGLNKEVVRQLSRRYPQLPRAVATGRVYDMNT     1122
SINV_P03317    KQSIPLTYHPADSARPVAHWDNSPGTRKYGYDHAIAAELSRRFPVFQL-AGKGTQLDLQT     1138
CHIK_Q8JUX6    KPLVSVYYA-------DNHWDNRPGGKMFGFNPEAASILERKYPFTKGKWNINKQICVTT     1124
CHIK_A6MH22    KPLVSVYYA-------DNHWDNRPGGKMFGFNPEAASILERKYPFTKGKWNINKQICVTT     1124
                  : :              **** *. . :* :    .   *.*::*          .     : *

VEEV_nsP2      588.........600.......610.......620.......630... ....640...646
VEEV_Q9WIJ1    GTLRNYDPRINLVPVNRRLPHALVLHHNEHPQSDFSSFVSKLKGRTVL-VVGEKLSVPGK     1181
VEEV_P27282    GTLRNYDPRINLVPVNRRLPHALVLHHNEHPQSDFSSFVSKLKGRTVL-VVGEKLSVPGK     1181
SINV_P03317    GRTRVISAQHNLVPVNRNLPHALVPEYKEKQPGPVKKFLNQFKHHSVLVVSEEKIEAPRK     1198
CHIK_Q8JUX6    RRIEDFNPTTNIIPANRRLPHSLVAEHRPVKGERMEWLVNKINGHHVLLVSGCSLALPTK     1184
CHIK_A6MH22    RRIEDFNPTTNIIPANRRLPHSLVAEHRPVKGERMEWLVNKINGHHVLLVSGYNLALPTK     1184
                   .   .    *::*.**.***:** .:.        .. ::.::: : ** *    .:   * *

VEEV_nsP2      647.....  .....660.......670.......680.......690.......700..
VEEV_Q9WIJ1    MVDWLSDR--PEATFRARLDLGIPGDVPKYDIIFVNVRTPYKYHHYQQCEDHAIKLSMLT     1239
VEEV_P27282    MVDWLSDR--PEATFRARLDLGIPGDVPKYDIIFVNVRTPYKYHHYQQCEDHAIKLSMLT     1239
SINV_P03317    RIEWIAPIGIAGADKNYNLAFGFPPQ-ARYDLVFINIGTKYRNHHFQQCEDHAATLKTLS     1257
CHIK_Q8JUX6    RVTWVAPLGVRGADYTYNLELGLPATLGRYDLVVINIHTPFRIHHYQQCVDHAMKLQMLG     1244
CHIK_A6MH22    RVTWVAPLGVRGADYTYNLELGLPATLGRYDLVVINIHTPFRIHHYQQCVDHAMKLQMLG     1244
                 : *::      *     .* :*:*    :**::.:*: * :: **:*** *** .*. *

VEEV_nsP2      705..710.......720.......730.......740.......750.......760.764
VEEV_Q9WIJ1    KKACLHLNPGGTCVSIGYGYADRASESIIGAIARQFKFSRVCKPKSSLEETEVLFVFIGY     1299
VEEV_P27282    KKACLHLNPGGTCVSIGYGYADRASESIIGAIARQFKFSRVCKPKSSLEETEVLFVFIGY     1299
SINV_P03317    RSALNCLNPGGTLVVKSYGYADRNSEDVVTALARKFVRVSAARPDCVSSNTEMYLIFRQL     1317
CHIK_Q8JUX6    GDSLRLLKPGGSLLIRAYGYADRTSERVICVLGRKFRSSRALKPPCVTSNTEMFFLFSNF     1304
CHIK_A6MH22    GDSLRLLKPGGSLLIRAYGYADRTSERVICVLGRKFRSSRALKPPCVTSNTEMFFLFSNF     1304
                .:    *:***: :  .****** ** :: .:.*:*     . :* .  .:**: ::*

                                                      nsP2*3
VEEV_nsP2      765. .770.......780.......790.794
VEEV_Q9WIJ1    DRKA-RTHNPYKLSSTLTNIYTGSRLHEAGCAPSYHVVRGDIATATEGVIINAANSKGQP     1358
VEEV_P27282    DRKA-RTHNPYKLSSTLTNIYTGSRLHEAGCAPSYHVVRGDIATATEGVIINAANSKGQP     1358
SINV_P03317    DNSRTRQFTPHHLNCVISSVYEGTR-DGVGAAPSYRTKRENIADCQEEAVVNAANPLGRP     1376
CHIK_Q8JUX6    DNGR-RNFTTHVMNNQLNAAFVGQ-ATRAGCAPSYRVKRMDIAKNDEECVVNAANPRGLP     1362
CHIK_A6MH22    DNGR-RNFTTHVMNNQLNAAFVGQ-ATRAGCAPSYRVKRMDIAKNDEECVVNAANPRGLP     1362
               *.    * .. : :.  :.  : *     .*.****:. * :**   *  ::****  * *

VEEV_Q9WIJ1    GGGVCGALYKKFPESFDLQPIEVGKARLVKGAAKHIIHAVGPNFNKVSEVEGDKQLAEAY     1418
VEEV_P27282    GGGVCGALYKKFPESFDLQPIEVGKARLVKGAAKHIIHAVGPNFNKVSEVEGDKQLAEAY     1418
SINV_P03317    GEGVCRAIYKRWPTSFTDSATETGTARMTVCLGKKVIHAVGPDFRKHPEAEALKLLQNAY     1436
CHIK_Q8JUX6    GDGVCKAVYKKWPESFKNSATPVGTAKTVMCGTYPVIHAVGPNFSNYSESEGDRELAAAY     1422
CHIK_A6MH22    GDGVCKAVYKKWPESFKNSATPVGTAKTVMCGTYPVIHAVGPNFSNYSESEGDRELAAAY     1422
               * *** *:**::* **  .   .*.*: .      :******:* :  *. * : *  **
```

```
VEEV_Q9WIJ1   ESIAKIVNDNNYKSVAIPLLSTGIFSGNKDRLTQSLNHLLTALDTTDADVAIYCRDKKWE    1478
VEEV_P27282   ESIAKIVNDNNYKSVAIPLLSTGIFSGNKDRLTQSLNHLLTALDTTDADVAIYCRDKKWE    1478
SINV_P03317   HAVADLVNEHNIKSVAIPLLSTGIYAAGKDRLEVSLNCLTTALDRTDADVTIYCLDKKWK    1496
CHIK_Q8JUX6   REVAKEVTRLGVNSVAIPLLSTGVYSGGKDRLTQSLNHLFTAMDSTDADVVIYCRDKEWE    1482
CHIK_A6MH22   REVAKEVTRLGVNSVAIPLLSTGVYSGGKDRLTQSLNHLFTAMDSTDADVVIYCRDKEWE    1482
              . :*. *.   . :**********:::..****  *** * **:* *****.*** **:*:

VEEV_Q9WIJ1   MTLKEAVARREAVEEICISDDSSVTEPDAELVRVHPKSSLAGRKGYSTSDGKTFSYLEGT    1538
VEEV_P27282   MTLKEAVARREAVEEICISDDSSVTEPDAELVRVHPKSSLAGRKGYSTSDGKTFSYLEGT    1538
SINV_P03317   ERIDAALQLKESVTELK----DEDMEIDDELVWIHPDSCLKGRKGFSTTKGKLYSYFEGT    1552
CHIK_Q8JUX6   KKISEAIQMRTQVELLD-----EHISIDCDVVRVHPDSSLAGRKGYSTTEGALYSYLEGT    1537
CHIK_A6MH22   KKISEAIQMRTQVELLD-----EHISIDCDIVRVHPDSSLAGRKGYSTTEGALYSYLEGT    1537
                :. *:  :  *  :       .  . * ::* :**.*.* ****:**:.*  :**:***

VEEV_Q9WIJ1   KFHQAAKDIAEINAMWPVATEANEQVCMYILGESMSSIRSKCPVEESEASTPPSTLPCLC    1598
VEEV_P27282   KFHQAAKDIAEINAMWPVATEANEQVCMYILGESMSSIRSKCPVEESEASTPPSTLPCLC    1598
SINV_P03317   KFHQAAKDMAEIKVLFPNDQESNEQLCAYILGETMEAIREKCPVDHNPSSSPPKTLPCLC    1612
CHIK_Q8JUX6   RFHQTAVDMAEIYTMWPKQTEANEQVCLYALGESIESIRQKCPVDDADASSPPKTVPCLC    1597
CHIK_A6MH22   RFHQTAVDMAEIYTMWPKQTEANEQVCLYALGESIESIRQKCPVDDADASSPPKTVPCLC    1597
              :***:* *:*** .::*   *:***:* * ***::.:**.****:.  :*:**.*:****

VEEV_Q9WIJ1   IHAMTPERVQRLKASRPEQITVCSSFPLPKYRITGVQKIQCSQPILFSPKVPAYIHPRKY    1658
VEEV_P27282   IHAMTPERVQRLKASRPEQITVCSSFPLPKYRITGVQKIQCSQPILFSPKVPAYIHPRKY    1658
SINV_P03317   MYAMTPERVHRLRSNNVKEVTVCSSTPLPKHKIKNVQKVQCTKVVLFNPHTPAFVPARKY    1672
CHIK_Q8JUX6   RYAMTPERVTRLRMNHVTSIIVCSSFPLPKYKIEGVQKVKCSKVMLFDHNVPSRVSPREY    1657
CHIK_A6MH22   RYAMTPERVTRLRMNHVTSIIVCSSFPLPKYKIEGVQKVKCSKVMLFDHNVPSRVSPREY    1657
               :******* **: ..   .: **** ****::* .***::*:: :**. :.*: :  *:*

VEEV_Q9WIJ1   LVETPPVDETPEPSAENQSTEGTPEQPPLITEDET-RTRTPEPIIIEEEEEDSISLLSDG    1717
VEEV_P27282   LVETPPVDETPEPSAENQSTEGTPEQPPLITEDET-RTRTPEPIIIEEEEEDSISLLSDG    1717
SINV_P03317   IEVPEQPTAPPAQAEEAPEVVATP---SPSTADNTSLDVTDISLDMDDSSEGSLFSSFSG    1729
CHIK_Q8JUX6   RPSQESVQEASTTTSLTHSQFD------LSV-D---GKILPVPSDLDADAPALEPALDDG    1707
CHIK_A6MH22   RSSPESAQEASTTTSLTHSQFD------LSV-D---GEILPVPPDLDADAPALEPALDDG    1707
                           :    .           . *             :: .          .*

VEEV_Q9WIJ1   PTHQVLQVEADIHGPPSVSSSSWSIPHASDFDVDSLSILDTLEGASVTSGATSAETNSYF    1777
VEEV_P27282   PTHQVLQVEADIHGPPSVSSSSWSIPHASDFDVDSLSILDTLEGASVTSGATSAETNSYF    1777
SINV_P03317   SDNS------------ITSMDSWSSGPSSL------EIVDRRQVVVADVHAV--------    1763
CHIK_Q8JUX6   AIHTLPSATGNL-----AAVSDWVMSTVPVAP------PRRRRGRNLTVTCDEREGNITP    1756
CHIK_A6MH22   ATHTLPSTTGNL-----AAVSDWVMSTVPVAP------PRRRRGRNLTVTCDEREGNITP    1756
                :                .: ..*                    .       .

VEEV_Q9WIJ1   AKSMEFLAR-PVPAP---------RTVFRNPPHPAPRTRT--PSLAPSRAC---------    1816
VEEV_P27282   AKSMEFLAR-PVPAP---------RTVFRNPPHPAPRTRT--PSLAPSRAC---------    1816
SINV_P03317   --------QEPAPIPPPRLKKMARLAAARKEPTPPASNSSESLHLSFGGVSMSLGSIFDG    1815
CHIK_Q8JUX6   MASVRFFRAELCPVVQETAETRDTAMSLQAPPSTATELSH--PPISFGAPS---------    1805
CHIK_A6MH22   MASVRFFRAELRPVAQETAETRDTATSLQAPPSTATELNH--PPISFGAPS---------    1805
                          *               :  *              :: .  .

VEEV_Q9WIJ1   ---------SRTSLVSTPPGVNRVITREELEALTPSRTPSRSVSRTSLVSNPPGVNRVIT    1867
VEEV_P27282   ---------SRTSLVSTPPGVNRVITREELEALTPSRTPSRSVSRTSLVSNPPGVNRVIT    1867
SINV_P03317   ETARQAAVQPLATGPTDVPMSFGSFSDGEIDELSRRVTES---EPVLFGSFEPGEVNSII    1872
CHIK_Q8JUX6   ---------E------TFPITFGDFNEGEIESLSS--------ELLTFGDFLPGEVDDLT    1842
CHIK_A6MH22   ---------E------TFPITFGDFNEGEIESLSS--------ELLTFGDFLPGEVDDLT    1842
                                 *     :.  *:: *:          .   :  .  **    :

                                      nsP3*4
VEEV_Q9WIJ1   REEFEAFV------------AQQQ----------------------------------    1879
VEEV_P27282   REEFEAFV------------AQQQXRFDAGAYIFSSDTGQGHLQQKSVRQTVLSEVVLER    1915
SINV_P03317   SSRSAVSFPLRKQRRRRRSRRTEYXLTGVGGYIFSTDTGPGHLQKKSVLQNQLTEPTLER    1932
CHIK_Q8JUX6   DSDWSTCS----------DTDDELRLDRAGGYIFSSDTGPGHLQQKSVRQSVLPVNTLEE    1892
CHIK_A6MH22   DSDWSTCS----------DTDDELRLDRAGGYIFSSDTGPGHLQQKSVRQSVLPVNTLEE    1892
               .   .                 :
```

```
VEEV_Q9WIJ1    ------------------------------------------------------------     1879
VEEV_P27282    TELEISYAPRLDQEKEELLRKKLQLNPTPANRSRYQSRKVENMKAITARRILQGLGHYLK     1975
SINV_P03317    NVLERIHAPVLDTSKEEQLKLRYQMMPTEANKSRYQSRKVENQKAITTERLLSGLRLYNS     1992
CHIK_Q8JUX6    VHEEKCYPPKLDEAKEQLLLKKLQESASMANRSRYQSRKVENMKATIIQRLKRGCRLYLM     1952
CHIK_A6MH22    VHEEKCYPPKLDEAKEQLLLKKLQESASMANRSRYQSRKVENMKATIIQRLKRGCRLYLM     1952

VEEV_Q9WIJ1    ------------------------------------------------------------     1879
VEEV_P27282    AE-GKVECYRTLHPVPLYSSSVNRAFSSPKVAVEACNAMLKENFPTVASYCIIPEYDAYL     2034
SINV_P03317    A-TDQPECYKITYPKPLYSSSVPANYSDPQFAVAVCNNYLHENYPTVASYQITDEYDAYL     2051
CHIK_Q8JUX6    SETPKVPTYRTTYPAPVYSPPINVRLSNPESAVAACNEFLARNYPTVSSYQITDEYDAYL     2012
CHIK_A6MH22    SETPKVPTYRTTYPAPVYSPPINVRLSNPESAVAACNEFLARNYPTVSSYQITDEYDAYL     2012

VEEV_Q9WIJ1    ------------------------------------------------------------     1879
VEEV_P27282    DMVDGASCCLDTASFCPAKLRSFPKKHSYLEPTIRSAVPSAIQNTLQNVLAAATKRNCNV     2094
SINV_P03317    DMVDGTVACLDTATFCPAKLRSYPKKHEYRAPNIRSAVPSAMQNTLQNVLIAATKRNCNV     2111
CHIK_Q8JUX6    DMVDGSESCLDRATFNPSKLRSYPKQHAYHAPSIRSAVPSPFQNTLQNVLAAATKRNCNV     2072
CHIK_A6MH22    DMVDGSESCLDRATFNPSKLRSYPKQHAYHAPSIRSAVPSPFQNTLQNVLAAATKRNCNV     2072

VEEV_Q9WIJ1    ------------------------------------------------------------     1879
VEEV_P27282    TQMRELPVLDSAAFNVECFKKYACNNEYWETFKENPIRLTEENVVNYITKLKGPKAAALF     2154
SINV_P03317    TQMRELPTLDSATFNVECFRKYACNDEYWEEFARKPIRITTEFVTAYVARLKGPKAAALF     2171
CHIK_Q8JUX6    TQMRELPTLDSAVFNVECFKKFACNQEYWEEFAASPIRITTENLTTYVTKLKGPKAAALF     2132
CHIK_A6MH22    TQMRELPTLDSAVFNVECFKKFACNQEYWEEFAASPIRITTENLTTYVTKLKGPKAAALF     2132

VEEV_Q9WIJ1    ------------------------------------------------------------     1879
VEEV_P27282    AKTHNLNMLQDIPMDRFVMDLKRDVKVTPGTKHTEERPKVQVIQAADPLATAYLCGIHRE     2214
SINV_P03317    AKTYNLVPLQEVPMDRFVMDMKRDVKVTPGTKHTEERPKVQVIQAAEPLATAYLCGIHRE     2231
CHIK_Q8JUX6    AKTHNLLPLQEVPMDRFTVDMKRDVKVTPGTKHTEERPKVQVIQAAEPLATAYLCGIHRE     2192
CHIK_A6MH22    AKTHNLLPLQEVPMDRFTVDMKRDVKVTPGTKHTEERPKVQVIQAAEPLATAYLCGIHRE     2192

VEEV_Q9WIJ1    ------------------------------------------------------------     1879
VEEV_P27282    LVRRLNAVLLPNIHTLFDMSAEDFDAIIAEHFQPGDCVLETDIASFDKSEDDAMALTALM     2274
SINV_P03317    LVRRLTAVLLPNIHTLFDMSAEDFDAIIAEHFKQGDPVLETDIASFDKSQDDAMALTGLM     2291
CHIK_Q8JUX6    LVRRLNAVLLPNVHTLFDMSAEDFDAIIAAHFKPGDTVLETDIASFDKSQDDSLALTALM     2252
CHIK_A6MH22    LVRRLNAVLLPNVHTLFDMSAEDFDAIIAAHFKPGDTVLETDIASFDKSQDDSLALTALM     2252

VEEV_Q9WIJ1    ------------------------------------------------------------     1879
VEEV_P27282    ILEDLGVDAELLTLIEAAFGEISSIHLPTKTKFKFGAMMKSGMFLTLFVNTVINIVIASR     2334
SINV_P03317    ILEDLGVDQPLLDLIECAFGEISSTHLPTGTRFKFGAMMKSGMFLTLFVNTVLNVVIASR     2351
CHIK_Q8JUX6    LLEDLGVDHSLLDLIEAAFGEISSCHLPTGTRFKFGAMMKSGMFLTLFVNTLLNITIASR     2312
CHIK_A6MH22    LLEDLGVDHSLLDLIEAAFGEISSCHLPTGTRFKFGAMMKSGMFLTLFVNTLLNITIASR     2312

VEEV_Q9WIJ1    ------------------------------------------------------------     1879
VEEV_P27282    VLRERLTGSPCAAFIGDDNIVKGVKSDKLMADRCATWLNMEVKIIDAVVGEKAPYFCGGF     2394
SINV_P03317    VLEERLKTSRCAAFIGDDNIIHGVVSDKEMAERCATWLNMEVKIIDAVIGERPPYFCGGF     2411
CHIK_Q8JUX6    VLEDRLTKSACAAFIGDDNIIHGVVSDELMAARCATWMNMEVKIIDAVVSQKAPYFCGGF     2372
CHIK_A6MH22    VLEDRLTKSACAAFIGDDNIIHGVVSDELMAARCATWMNMEVKIIDAVVSLKAPYFCGGF     2372

VEEV_Q9WIJ1    ------------------------------------------------------------     1879
VEEV_P27282    ILCDSVTGTACRVADPLKRLFKLGKPLAADDEHDDDRRRALHEESTRWNRVGILSELCKA     2454
SINV_P03317    ILQDSVTSTACRVADPLKRLFKLGKPLPADDEQDEDRRRALLDETKAWFRVGITGTLAVA     2471
CHIK_Q8JUX6    ILHDIVTGTACRVADPLKRLFKLGKPLAAGDEQDEDRRRALADEVVRWQRTGLIDELEKA     2432
CHIK_A6MH22    ILHDTVTGTACRVADPLKRLFKLGKPLAAGDEQDEDRRRALADEVIRWQRTGLIDELEKA     2432

VEEV_Q9WIJ1    ------------------------------------------             1879
VEEV_P27282    VESRYETVGTSIIVMAMTTLASSVKSFSYLRGAPITLYG---             2493
SINV_P03317    VTTRYEVDNITPVLLALRTFAQSKRAFQAIRGEIKHLYGGPK             2513
CHIK_Q8JUX6    VYSRYEVQGISVVVMSMATFASSRSNFEKLRGPVVTLYGGPK             2474
CHIK_A6MH22    VYSRYEVQGISVVVMSMATFASSRSNFEKLRGPVITLYGGPK             2474
```

Figure S2. Electron density of VEEV nsP2pro active site. Representative fit of the active site including the residues of N-terminal region (K473-C477) and the catalytic residues (C477, H546) to the final electron density map (blue, 1.46 Å resolution, contoured at 1.0 r.m.s.d level).

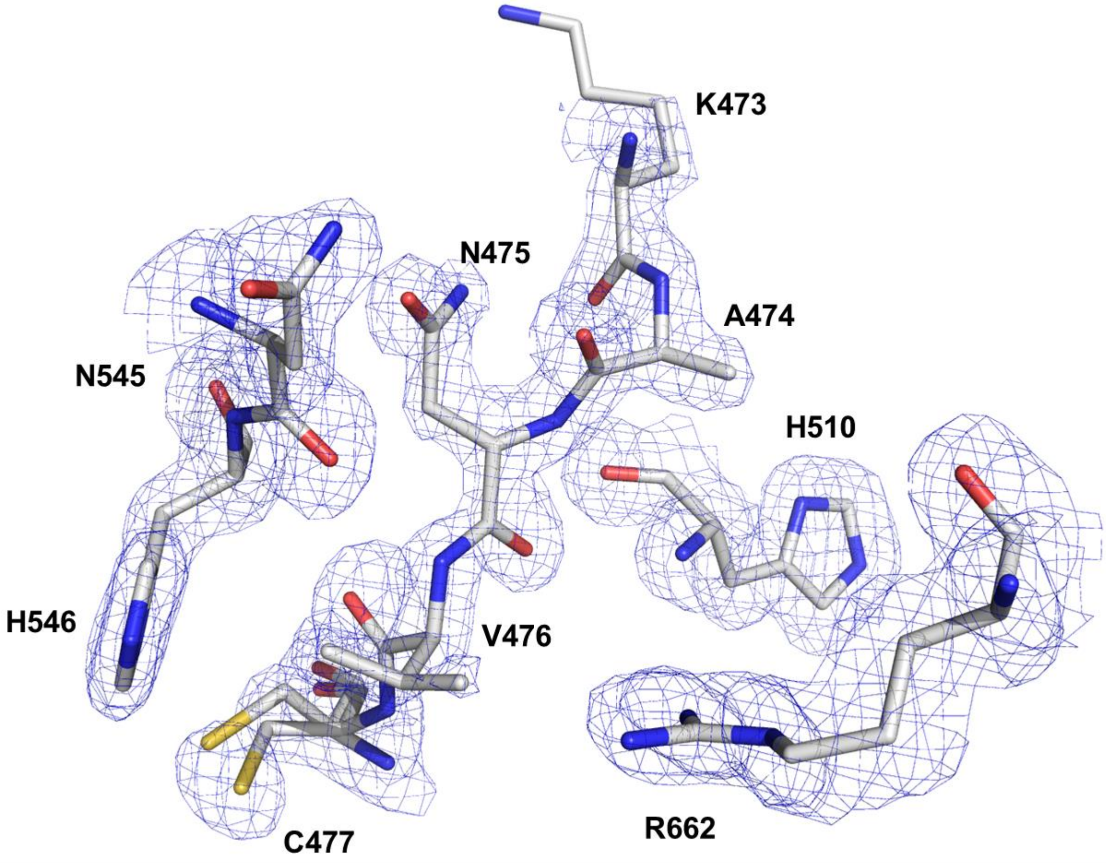

Figure S3. Conformation of the Y764-Y774 loop of the SAM MTase domain in crystal structures of VEEV nsP2pro. (**a**) Alignment of VEEV nsP2pro crystal structures is shown (PDB IDs: 8DUF, 6BCM, 5EZQ, and 2HWK). The overall structures are represented, the mutated K741 and K767 residues are labelled. (**b**) The Y764-Y774 loop of the SAM MTase domain is shown. Cyan coloured dashed line shows the loop for which electron density was not visible (PDB ID: 8DUF).

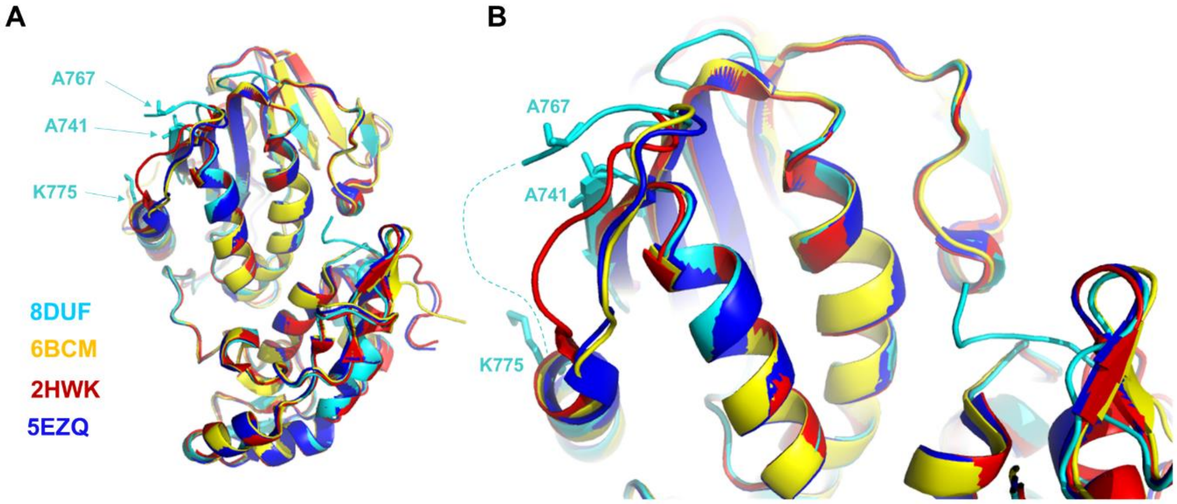

Figure S4. Active site residues. Figure was prepared based on the crystal structure of VEEV nsP2pro (PDB ID: 8DUF). A' and B' conformers are also represented, C477, N475, H546, and R662 residues are labelled. Arrows indicate distances which were measured between the center of mass of the residues. The values are plotted in **Figure 10**.

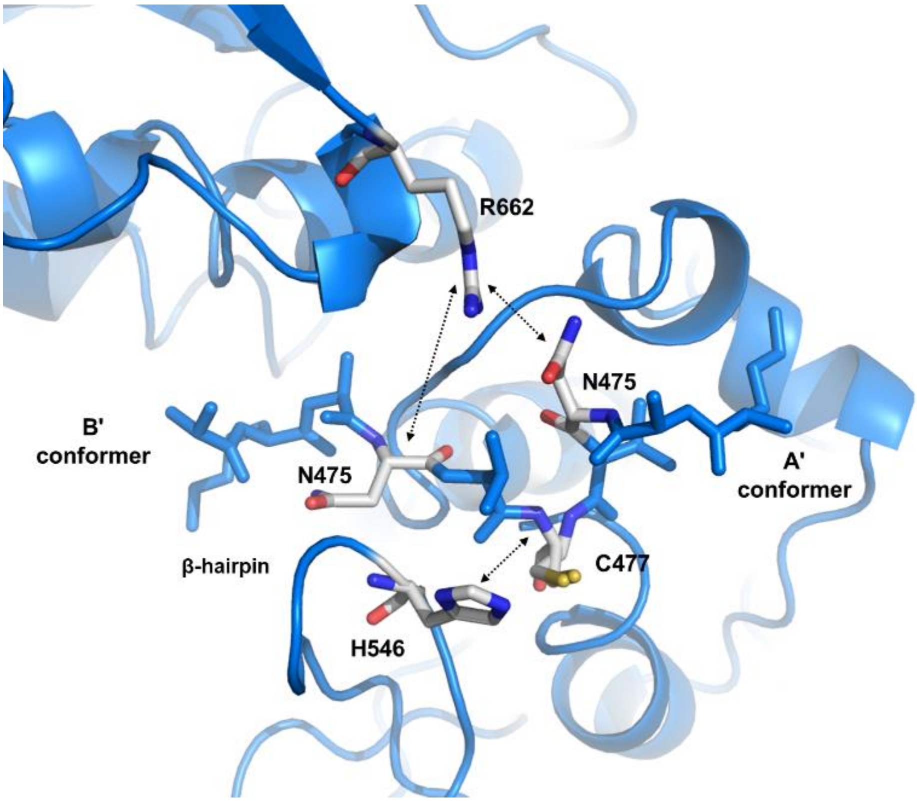

Supplement: Supplementary [file NIHMS1942432-supplement-Supplementary.pdf]
